# Cluster randomized trials of individual-level interventions were at high risk of bias

**DOI:** 10.1016/j.jclinepi.2021.06.021

**Published:** 2021-10

**Authors:** Christina Easter, Jennifer A. Thompson, Sandra Eldridge, Monica Taljaard, Karla Hemming

**Affiliations:** a Institute of Applied Health Research, University of Birmingham, Birmingham, UK; b Department of Infectious Disease Epidemiology, London School of Hygiene and Tropical Medicine, London, UK; c Centre for Clinical Trials and Methodology, Queen Mary University of London, London; d Clinical Epidemiology Program, Ottawa Hospital Research Institute, Ottawa, Ontario, Canada; e School of Epidemiology and Public Health, University of Ottawa, Ottawa, Canada

**Keywords:** Cluster randomized trials, Risk of bias, Individual-level interventions, Selection bias

## Abstract

**Objectives:**

To describe the prevalence of risks of bias in cluster-randomized trials of individual-level interventions, according to the Cochrane Risk of Bias tool.

**Study design and setting:**

Review undertaken in duplicate of a random sample of 40 primary reports of cluster-randomized trials of individual-level interventions.

**Results:**

The most common reported reasons for adopting cluster randomization were the need to avoid contamination (17, 42.5%) and practical considerations (14, 35%). Of the 40 trials all but one was assessed as being at risk of bias. A majority (27, 67.5%) were assessed as at risk due to the timing of identification and recruitment of participants; many (21, 52.5%) due to an apparent lack of adequate allocation concealment; and many due to selectively reported results (22, 55%), arising from a mixture of reasons including lack of documentation of primary outcome. Other risks mostly occurred infrequently.

**Conclusion:**

Many cluster-randomized trials evaluating individual-level interventions appear to be at risk of bias, mostly due to identification and recruitment biases. We recommend that investigators carefully consider the need for cluster randomization; follow recommended procedures to mitigate risks of identification and recruitment bias; and adhere to good reporting practices including clear documentation of primary outcome and allocation concealment methods.

## Highlights

• Due to the risks of identification and recruitment bias, opting for a cluster design when individual randomization would be feasible needs a strong justification. Concerns around contamination are unlikely to be acceptable justifications; although estimation of indirect effects might be.
• When cluster randomization is adopted, we recommend that authors provide a clear justification for the choice of cluster randomization and clearly outline strategies to mitigate increased risks of bias. This should include identification and recruitment by someone blind to the treatment allocation and minimal or objective individual-level eligibility criteria.
• Other good conduct procedures which are routinely implemented in individually randomized trials should be followed. These include implementation of the randomization using an accepted method of allocation concealment, for example, by using an independent statistician to generate the allocation sequence; blind outcome assessment when outcomes are subjective; and clear pre-specification (in a protocol or trial registration site) of the primary outcome including primary assessment time and method of primary analysis.
• All these aspects should be clearly reported as per CONSORT guidelines. To ensure particular clarity around identification and recruitment, authors should also provide a timeline-cluster diagram.

**What is New?**

**Current knowledge**

Cluster-randomized designs are known to be at increased risk of bias compared to the individually randomized design.

To date, these risks of bias have not been specifically documented in cluster trials of individual-level interventions where individual randomization would in theory be feasible.

**Findings from our study**

In our review of a random sample of 40 cluster-randomized trials of individual-level interventions, we found that all but one was at risk of bias.

Trials were at risk of bias across multiple domains, but a prominent source was identification and recruitment bias.

**Recommendations**

Due to the risks of identification and recruitment bias, opting for a cluster design when individual randomization would be feasible needs a strong justification. Concerns around contamination are unlikely to be acceptable justifications; although estimation of indirect effects might be.

When cluster randomization is used, with post randomisation recruitment, identification and recruitment should be undertaken by someone blind to the treatment allocation with minimal or objective individual-level eligibility criteria.

## 1 Introduction

In individually randomized trials, patients are randomly allocated to different interventions, henceforth referred to as treatment or control conditions. Rather than randomizing individual patients, cluster-randomized trials randomize entire clusters (such as wards, schools or social groups) to treatment or control conditions [12], [27], [33], [34]. Cluster-randomized trials can be used to evaluate different types of interventions, sometimes delivered at the level of the entire cluster (cluster-level interventions), sometimes delivered at the level of the health care professionals (professional-level intervention), sometimes delivered directly to individual patients (individual-level intervention) and sometimes a mixture [8,9]. Cluster-level and professional-level intervention necessarily require cluster randomization.

Cluster-randomized designs are known to be at increased risk of bias compared to the individually randomized design [1], [2], [7], [10], [13], [16], [28], [37]. These risks of bias often challenge the strength of the evidence generated from this design and downgrade the quality of evidence that they contribute to systematic reviews [22]. Risks of bias in randomized trials have been carefully described in the Cochrane systematic review Risk of Bias tool (RoB2.0) [19] and an adaptation of the main guidance has been developed for cluster trials. Recruitment and identification biases are a unique source of bias under cluster randomization, with trials being particularly vulnerable to this bias when it is necessary to identify or recruit individuals into the study after randomization [1,11]. For example, to evaluate a pharmacological intervention without blinding and with randomization at the level of a village, if recruitment occurs after randomization, then the decision to participate (or not) might be affected by knowledge that they will receive the active intervention (or not). Such beliefs can affect outcomes, and therefore may bias the study's estimates of the between-group effect. Recommendations suggest that to avoid or reduce these risks, trials adopt broad eligibility criteria at the level of the individual and, if participants cannot be identified and recruited prior to randomization, identification and recruitment of participants is by someone who is blind to the cluster allocation [11,14,16].

Whilst there may be good reasons for adopting cluster randomization including to avoid contamination (e.g., individuals in the control condition being exposed to interventions) and for logistical simplicity (e.g., to simplify the fieldwork by having only one type of intervention in a particular cluster or geographical area) [35], individual-level interventions could, in theory, be evaluated with an individually randomized trial. Whilst other reviews have documented risks of bias in cluster trials more generally, none have documented risks of bias in cluster trials of individual-level intervention where individual randomization would in theory be feasible. Here, we report the results of a review of the risks of bias in contemporary primary reports of cluster-randomized trials of individual-level interventions. Our objectives were to (i) identify the prevalence of key risks of bias in cluster-randomized trials of individual-level interventions; (ii) to describe prevalence of design features associated with increased risks of bias and (iii) formulate design recommendations to avoid such risks. We also describe the reliability of the two independent assessments of risk of bias.

## 2 Methods

### 2.1 Scope of review

We used a convenience sample of trials identified in a previously published review of cluster trials of individual-level interventions published in the interval from 2007 to 2016 [31]. In brief, the review included primary reports of cluster-randomized trials of individual-level therapeutic interventions conducted in Canada, USA, European Union, Australia, and Low- or Middle-Income Country (LMIC) and published in English. Individual-level interventions were defined as any intervention that is aimed solely at the individual; thus, we excluded evaluations of cluster-level or professional-level interventions and evaluations where these types of intervention were included alongside an individual-level intervention. Therapeutic interventions were defined broadly as medicinal, clinical or surgical based interventions (see [31] for a full definition). Full text articles were screened in a random sequence until a sample size of 40 was achieved.

### 2.2 Justification for scope

We used an existing database of primary reports of individual-level cluster-randomized trials for logistical reasons: screening and review of a very large number of citations from the general medical literature to isolate primary reports of cluster trials with exclusively individual-level interventions is a substantial undertaking; and furthermore, using this existing sampling frame allowed us to obtain a random sample of such trials. Including individual-level interventions only, whilst narrowing scope of generalizability, allows us to meet our objective of evaluating risk of bias in situations where a theoretical alternative is the individually randomized design. Focusing on therapeutic interventions targets our finding to the evaluation of interventions intended to bring about improvements in health.

### 2.3 Data abstraction process

Data were abstracted from the full trial reported. We additionally searched the full trial reports to identify any reference to study protocols or statistical analysis plans (which sometimes included additional study information such as patient information and consent forms) and searched for trial registration documentation for each included study by using any trial registration reported in the text, or using google searches to identify any registration. All data was abstracted by one reviewer (CE) and independently and in duplicate by a second randomly allocated reviewer (KH, CK, JT or JM). After both assessments were completed, disagreements were identified, and a consensus (henceforth referred to as the joint assessment) reached by discussion. Where necessary, a third reviewer was consulted to reach agreement (KH or JT). The data capture was electronic (using RedCap). Study reports were randomly sorted before data abstraction.

### 2.4 Data abstracted on general characteristics of trials

We abstracted the following trial characteristics: publication year; country of conduct; type of cluster; rationale for cluster design; trial design (parallel, factorial, cross-over, stepped-wedge); number of clusters randomized; average (realized) cluster size. We also extracted whether a trial protocol, statistical analysis plan or trial registration were available because in the absence of such documentation, it is impossible to determine whether the primary outcome was pre-specified. We extracted the number of eligibility criteria at the participant level as more eligibility criteria increases the likelihood of differential inclusion [14]. We also classified each trial based on whether it was reported that an independent person conducted the randomization as this is an indicator of concealment of the randomization process. Additionally, we extracted our assessment of whether the outcome was subjective or objective.

### 2.5 Data abstracted on risk of bias

For each study report, reviewers were provided with a detailed risk of bias assessment form (Supplementary Material 1). This risk of bias assessment aimed to assess the risk of bias for each of the five domains in the RoB2.0 tool (Table 1). These domains are (i) bias arising from the (a) randomization process and (b) the timing of identification and recruitment of participants in relation to the timing of the randomization; ii) bias due to deviations from the intended intervention; iii) bias due to missing outcome data; iv) bias due to the measurement of the outcome; and v) bias due to the selection of the reported result.

**Table 1:** Summary and description of Risks of Bias in cluster-randomized trials as documented in RoB2.0 adaption for cluster trials

Domain	Description
Domain 1a: Bias arising from the randomization process	Randomization refers to the process of allocating clusters to arms. Biases can arise if this allocation is not random or is not adhered to (at the level of the cluster).
Domain 1b: Bias arising from identification or recruitment of participants within clusters	When identification and recruitment of participants occurs with knowledge of the treatment allocation this can lead to differential recruitment and identification between treatment conditions.
Domain 2: Bias due to deviations from intended interventions	Trials which intend to measure the effect of offering treatment in everyday practice are unlikely to be conducted with blinding of the participant to allocated treatment. Deviations from the intended intervention can occur if those in the control condition receive the intervention condition (or vice versa). This is sometimes referred to as contamination or performance bias.
Domain 3: Bias due to missing outcome data	Missing outcome data often occurs in randomized trials. Where the missingness is differential across treatment conditions, this can cause bias. Missingness can be differential across treatment conditions even when the proportion missingness is similar across conditions (for example when missingness is dependent on prognostic factors).
Domain 4: Bias in measurement of the outcome	Trials in which the treatment status is known by those assessing outcomes might be at risk of bias because of (subconscious) assessments of outcomes being preferential in one treatment condition. Outcomes which are objective (e.g., mortality) will be at reduced risk of this bias. This is sometimes referred to as outcome assessment bias.
Domain 5: Bias in selection of the reported result	Trials which do not pre-specify the primary outcome, along with primary assessment time, or clear method of analysis (including factors for adjustment) are at risk of selecting positive outcomes at the time of reporting.

In the RoB2.0 tool, under an extension for cluster trials (accessed May 2019; dated October 20, 2016), these risks are identified by a series of *signalling questions* with an extensive set of *elaborations* providing extensive detail about how to answer the signal questions [19]. To avoid having to refer back to the extensive elaborations, we mapped the *signalling questions* from RoB2.0 and their associated set of *elaborations* onto a set of data abstraction *items* (Supplementary Material 2). As an illustrative example, domain 1a is “Bias arising from the randomization process” and one of the three signalling questions for this domain is “Was the allocation sequence random?” and the associated question on our mapped data abstraction item was “How was the randomization of clusters to allocated treatment(s) conducted? (Tick all that apply)”. Following the reasoning outlined in the elaboration of RoB2.0, trial reports which were identified as using one of the random methods of allocation defined in the explanatory material were then classified as using a random allocation method. Another associated signalling question is “Was the allocation sequence concealed until clusters were enrolled and assigned to interventions?” and the associated data abstraction items were “Who conducted the randomization?” and “How was the randomization allocation of clusters concealed?” Again, following the elaboration outlined in RoB2.0, study reports which reported the randomization to be by someone independent, or using a trials unit, or using some other acceptable concealment mechanism, such as internet-based randomization or sealed envelopes, were classified as having a concealed allocation process.

From this we obtained for each signalling question an assessment of “yes”, “no”, and “no information” (the independent assessment stage also included the option “unclear” but this option was not retained at the joint assessment; we did not use the classification of “probably yes” or “probably no”). We followed the RoB2.0 mapping from these signalling questions to risks of bias assessment for each domain to classify each trial under each domain as “low risk of bias”, “some concerns” or “high risk of bias” (again at the independent assessment stage the option “unclear” was also included). Of note, this means that no trials were assessed as at unclear risk as this is no longer a domain in the RoB2.0 tool (any assessments of “no information” are mapped to the relevant category following the RoB2.0 mapping). Finally, again following RoB2.0 we created an overall study assessment of risk of bias: a study is judged at high risk of bias if it is assessed at high risk in at least one domain or some concerns for multiple domains; low risk of bias if it is assessed as low risk in all domains; and some concerns otherwise. A small number of assumptions were made along the way and these are noted in the table footnotes and in the results section below.

### 2.6 Statistical analysis

We describe the assessment of risk of bias (based on the consensus / joint agreement) for all domains and signalling questions, using simple descriptive statistics (numbers and percentages). We also describe the reliability of the independent assessments (not the final joint assessment), by computing the percentage agreement (including raw percentage agreement and the Gwet's AC value [15,36]) between the two independent assessments for each broad domain and for each of the signalling questions. Reliability was computed across a non-ordinal four-point scale for both risk of bias (high risk of bias / some concerns /low risk of bias / unclear); and across signalling questions (“yes”, “no”, “no information”, “unclear”). Gwet's AC statistic was unweighted due to the non-ordinal categories for the signalling questions but weighted for the risk of bias (with the penalization set to thirds: low penalization set to 2/3 for high-some concerns, low-some concerns and anything-unclear; and high penalization set to 1/3 for high-low concerns).

## 3 Results

### 3.1 Study characteristics

Full information on the random sample selection can be found elsewhere [31], in brief the search identified 10,014 potential studies (after removal of duplicates), of which 3,097 were not excluded at the abstract screen. Of these 1,190 underwent a full text screen until 40 were identified as meeting the eligibility criteria. A description of the 40 trials is provided in Table 2. The trials were conducted between 2007 and 2016 and covered a range of settings including LMICs (21, 52.5%), Canada / USA (7, 17.5%) and Europe (11, 27.5%) amongst others; the most common reported reason for adopting cluster randomization was avoiding contamination (17, 42.5%) and practical reasons (14, 35%), and 10% (10 trials) did not report the rationale for cluster design. The most common form of cluster was a residential area (15, 37.5%) or hospital / nursing home / clinic (15, 37.5%); the median number of clusters included in each study was 24 (inter-quartile range, IQR: 12–49.5); the median cluster size was 114 (IQR: 35–456); and most designs were parallel (28, 70%). Only a minority of trials had an accessible protocol paper or statistical analysis plan (16, 40%), although most were registered on a trial registration site (33, 82.5%). A sizeable minority (6, 15%) had no documentation available to verify any pre-specification, for example of the primary outcome. Few used an independent statistician to implement the randomization (11, 27.5%). The majority had more than three eligibility criteria at the level of the individual (24, 60%). Most studies (30, 75%) were assessed to have objective primary outcome.

**Table 2:** Characteristics of trials included in review (N = 40)

Characteristic	n (%)
Publication year2007–20102011–20132014–2016	9 (22.5)20 (50.0)11 (27.5)
Country of study conductCanada and/or USAUnited Kingdom and/or EUAustraliaLMICs	7 (17.5)11 (27.5)1 (2.5)21 (52.5)
Type of clusterResidential areasPrimary care practicesIndividual health professionalsHospitals, nursing homes, medical clinics or ICUsOther	15 (37.5)4 (10)2 (5)15 (37.5)4 (10)
Rationale for cluster design^b^Avoid contaminationPractical reasonsCluster level analysisNo justificationOther	17 (42.5)14 (35)2 (5)10 (25)10 (25)
Trial designParallel armFactorialCross-overStepped wedge	28 (70)3 (7.5)6 (15)3 (7.5)
Pre-specification documentation availabilityAccessible protocol paper or SAPTrial registrationNeither protocol paper nor trial registration	16 (40)33 (82.5)6 (15)
Randomization by independent statistician	11 (27.5)
Number of eligibility criteria at the individual level<3>=3	16 (40)24 (60)
Number of clusters^a^Median (IQR)	24 [12 – 49.5]
Average cluster size^a^Median (IQR)	114 [35 – 456]
Outcome objectiveYesNo	30 (75)10 (25)

*Abbreviations:* ICU, intensive care unit; IQR, interquartile range; LMIC, low- or middle-income country; SAP, statistical analysis plan

a Numbers refer to realized numbers as opposed to those planned in any sample size calculation for example (i.e., the number of clusters randomized and the number of participants on whom baseline measures were taken)

b categories not mutually exclusive.

### 3.2 Broad assessment of risk of bias

**Fig. 1:**
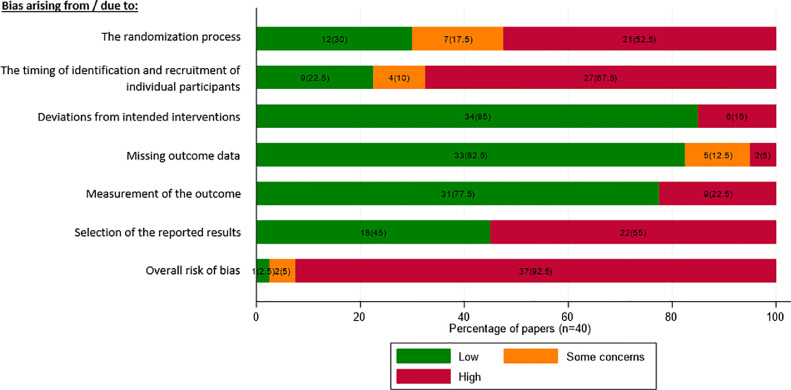
Percentage of papers in each risk category across the broad domains of risk.

Overall, all but three of the trials were assessed as at high risk of bias and only one was assessed at low risk of bias (Table 3, Fig. 1). Most trials were assessed as high risk on one (9, 22.5%) or two (14, 35%) domains; with a smaller number being assessed as high risk on up to 4 (6, 15%) or 5 (1, 2.5%) domains. Breaking down these assessments into finer categories (Supplementary Tables 1a to 5) helps identify the design features associated with these risks of bias. We next consider each domain separately.

**Table 3:** Risk of bias assessment by broad domains of risk

Domain	Level of Risk	n (%)	Reliability between reviewers	
		n = 40	Gwet's AC (95% CI)	% Agreement
1a - Bias arising from the randomization process	Low risk	12(30)	0.46(0.20,0.72)	50
	Some concerns	7(17.5)		
	High risk	21(52.5)		
				
1b - Bias arising from the timing of identification and recruitment of individual participants	Low risk	9(22.5)	0.59(0.37,0.81)	62.5
	Some concerns	4(10)		
	High risk	27(67.5)		
				
2 - Bias due to deviations from intended interventions	Low risk	34(85)	0.85(0.74,0.96)	75
	Some concerns	0(0)		
	High risk	6(15)		
				
3 - Bias due to missing outcome data	Low risk	33(82.5)	0.77(0.62,0.92)	67.5
	Some concerns	5(12.5)		
	High risk	2(5)		
				
4 - Bias in measurement of the outcome	Low risk	31(77.5)	0.79(0.64,0.95)	75
	Some concerns	0(0)		
	High risk	9(22.5)		
				
5 - Bias in selection of the reported results	Low risk	18(45)	0.44(0.19,0.70)	57.5
	Some concerns	0(0)		
	High risk	22(55)		
				
Overall risk of bias judgement^a^	Low risk	1(2.5)	0.92(0.85,0.99)	82.5
	Some concerns	2(5)		
	High risk	37(92.5)		
				
Number of domains at high risk	0^b^	3 (7.5)		
	1	9 (22.5)		
	2	14 (35)		
	3	7 (17.5)		
	4	6 (15)		
	5	1 (2.5)		

a Overall risk of bias judgement: low risk of bias is defined as all domains at low risk of bias; some concerns are defined as at least one domain has some concerns but does not include any high risk of bias for any domain; and high risk of bias is defined as high risk of bias in at least one domain or some concerns for multiple domains

b 0 domains at risk includes 1 at low risk and 2 with some concerns (overall risk).

*Domain 1a bias arising from the randomization process:* Around half of the trials (21, 52.5%) were assessed as being at high risk of bias due to the randomization process. Whilst all were assessed to use a random method to allocate clusters to treatment conditions, many (21, 52.5%) were assessed as not having concealed the allocations (i.e., not clearly reporting randomization by someone independent, or using a trials unit, or not using some acceptable concealment mechanism, such as internet-based randomization or sealed envelopes). Most trials (30, 75%) did not report any cluster-level characteristics to allow any assessment of balance of the randomization process.

*Domain 1b bias arising from identification or recruitment of participants within clusters:* A large majority of the trials (27, 67.5%) were assessed as at risk of bias due to the timing of identification and recruitment of participants. Most trials (35, 87.5%) were assessed as identifying or recruiting participants after randomization and most (27, 67.5%) were assessed to include participants in such a way that selection could have been affected by knowledge of the intervention. As shown in Supplementary Table 6, this is because many trials both recruited participants post randomization and those recruiting participants were not reported to be blind to the intervention. In some trials (15, 37.5%), we identified baseline imbalances that suggest differential identification or recruitment of individual participants between arms.

*Domain 2 bias due to deviations from intended interventions:* Most trials (34, 85%) were at low risk of bias due to deviations from the intended interventions. However, in a large number of trials, we deemed that participants were aware that they were in a trial (27, 67.5%) and aware of their assigned intervention (20, 50%), as did trial personnel (34, 85%). Despite this, only a minority of trials (8, 20%) were assessed as showing evidence of deviations from the intended intervention beyond what would be expected in usual practice; and in only a few trials (6, 15%) were these deviations from intended intervention unbalanced between groups and assessed as likely to have affected the outcome (Supplementary Table 7). Here we assumed that a deviation of the intended intervention occurred if more than 10% of the participants were reported not to have received the intended intervention condition. In all trials, most clusters and participants were reported to be analyzed according to randomization (i.e., by intention to treat).

*Domain 3 bias due to missing outcome data:* Most trials (33, 82.5%) were assessed as at low risk of bias due to missing outcome data, mostly because missing data arose infrequently: only in a small number of trials (9, 22.5%) was the outcome data unavailable for more than 10% of participants. In a small number of cases (4, 10%) outcome data were deemed to be differential across treatment arms.

*Domain 4 bias in measurement of the outcome:* Most of the trials were assessed as being at low risk of bias due to measurement of the outcome (31, 77.5%), although some (9, 22.5%) were assessed as being at high risk of bias. Whilst in almost all trials (36, 90%), outcome assessors were aware the trial was taking place and in many (26, 65%) they were aware of the intervention received by the participant, because most outcomes were assessed as objective (30, 75%, Table 2) this lack of blinding was assessed as inconsequential (for outcome assessment).

*Domain 5 bias in selection of the reported result:* A large proportion of the trials (22, 55%) were assessed as at high risk of bias in the selection of the reported result, and this arose due to multiple reasons. For a sizeable number of trials (14, 35%) the primary outcome was not clearly defined, either because the outcome itself was not clearly defined (7, 17.5%) in any of the trial registration database, study protocol, or methods section of the main trial report, or, because the primary assessment time was not clearly defined (9, 22.5%). For a few trials it was not stated if the primary analysis would be adjusted or unadjusted for covariates (6, 15%). Almost all trials reported the scale the primary outcome would be measured on, and how any binary variables would be categorised, but some were assessed as not having a plan for how they would handle missing data despite having missing data (9, 22.5%).

### 3.3 Reliability of independent assessments

The raw percentage agreement between the independent assessments were calculated for each signalling question, domain and overall risk of bias for each paper (Table 3 and Supplementary Table 8). For the overall assessment of each study the agreement was high (Gwet's AC: 0.92 95% CI: 0.85,0.99), but this varied across the different domains: agreement was 0.46 (95% CI: 0.20,0.72) for domain 1a (randomization process); 0.59 (95% CI: 0.37,0.81) for domain 1b (identification and recruitment process); 0.85 (95% CI: 0.74,0.96) for domain 2 (deviations from intended interventions); 0.77 (95% CI: 0.62,0.92) for domain 3 (missing outcome data); 0.79 (95% CI: 0.64,0.95) for domain 4 (measurement of the outcome) and 0.44 (95% CI: 0.19,0.70) for domain 5 (selection of reported result).

Particular signalling questions which had strikingly low reliability included whether the allocation was concealed from the clusters at randomization (0.41, 95% CI: 0.19,0.62); whether the selection of individual participants was likely affected by knowledge of the intervention (0.56, 95% CI: 0.36, 0.76); whether there were baseline imbalances across individual-level characteristics (0.53, 95% CI: 0.33,0.73); whether participants were aware of their assigned intervention (0.53, 95% CI: 0.33,0.74); whether proportions of missing data were similar across interventions (0.59, 95% CI: 0.40,0.78); as well as selection of reporting, for both the outcome (0.58, 95% CI: 0.36,0.79) and selected analysis (0.52, 95% CI: 0.30,0.74).

## 4 Discussion

### 4.1 Summary of findings

In our review of a random sample of 40 cluster-randomized trials of individual-level interventions, we found that all but one was at risk of bias. Trials were at risk of bias across multiple domains, but a prominent source was identification and recruitment bias. We found that the vast majority of cluster-randomized trials of individual-level interventions identify or recruit research participants after randomization of clusters to treatment conditions and fail to report use of any strategies to prevent identification and recruitment bias. In many it was deemed possible that selection of individual participants could be affected by knowledge of the intervention; with some showing evidence of baseline imbalance on individual-level characteristics across treatment arms.

We identified other possible risks of bias not necessarily specific to the use of cluster randomization. For example, many trials were assessed as not implementing randomization in a way that is clearly concealed. This is something which is easily correctable by use of an independent statistician or other acceptable concealed randomization method. Other risks of bias included a failure to clearly specify or document the primary outcome or primary assessment time: a small minority of trials neither publish a protocol paper (or statistical analysis plan) nor pre-register the trial on a trial registration database. In these trials, there is no possible way to verify any pre-specified primary outcome and these trials will be at risk of selective reporting. Related to this, many trials were assessed as not clearly documenting other features of their outcomes (such as primary assessment time) and analysis plan. Some studies were assessed as being at risk of bias due to measurement of the outcome; this might be surmountable in some trials by using blind outcomes assessors when outcomes are subjective.

The one trial identified as low risk of bias was a trial of skin cleansing wipe in new-born babies with a placebo control [32]. The placebo control helps minimize risk of bias in most domains: for example, despite the use of post-randomization identification and recruitment, there is no risk of identification and recruitment bias because the placebo control ensures recruitment is blind to the intervention condition. Furthermore, the outcome assessment is blinded (and in this trial also happened to be objective, namely mortality).

### 4.2 Limitations

We used a convenience sample of trials identified in another review. This means we have assessed risk of bias in a relatively small sample of 40 trials over an extended period of time between 2007 and 2016. Both reporting and conduct might have improved in recent years with the use of the CONSORT statement extension for cluster randomized trials [5], but most evaluations of reporting and conduct suggest that improvements are minimal at best [6]. Moreover, these trials are a true random sample of cluster-randomized trials of individual-level interventions across all journals, which should mean these results are representative of other cluster-randomized trials of similar types of interventions. We opted to use this sample as identifying a true random sample of cluster trials of individual-level interventions is very labor intensive and beyond our scope. Rather than taking a random sample, as much less labor-intensive search strategy would have been to focus on specific journals, such as high impact journals, but this tends to underestimate the scale of any problem.

Our assessment of bias, by following RoB2.0, assesses in part theoretical risk as well as manifestations of actual risk such as imbalance across trial arms [19]. We also used an earlier version of this tool (downloaded in May 2019, dated October 20, 2016) and there have subsequently been several minor revisions (March 2021). Assessment of risk of bias in both randomized and non-randomized studies is important, and despite availability of multiple tools, can be difficult. Others have shown that the reliability of assessments based on reviewing trial reports might be low for assessments which involve subjectivity [17,23,24]; and our results are consistent with these findings: independent assessments showed low reliability for questions which involve some subjectivity (e.g., whether there was any imbalance) and were generally lower than those that might be considered more objective (e.g., was the study randomized).

Whilst we assessed the reliability of the two independent assessments of bias, it is important to note that assessments of reliability should not be considered an assessment of reliability of the RoB2 tool. To assess the reliability of the RoB2 tool it is necessary to assess the reliability of the joint assessments and to this end it would be necessary to repeat the two independent assessments and their discussion, so as to obtain two joint assessments. The reliability of the joint assessment is expected to be higher than the reliability of the independent assessments as the joint consensus involved extensive discussion process to reconciliate individual assessments. We therefore do not suggest that our assessment is an assessment of the reliability of the RoB2 tool, despite others having suggested reliability between two independent measures can assess the reliability of RoB2 [25]. Nonetheless domains or signalling questions with low agreement might be indicative of domains or signalling questions which are less clearly amenable to an assessment of bias than those with higher agreement, and this might be translate more generally when others are using the RoB2 tool to assess risk of bias within the context of a review. Low reliability might either reflect poor reporting of the relevant items in the primary paper or the requirement to make a subjective assessment and in both cases, it might be necessary for reviewers to make assumptions.

By necessity we made assumptions. For example, not all trials clearly reported whether participants were actively recruited into the study, here we assumed that any mention of “consent” equated to active recruitment. In many trials it was difficult to identify if recruitment occurred post randomization. Again, here we made assumptions, for example, in an acute setting such as the intensive care unit, we assumed patient accrual had to occur post randomization; or when the recruitment period was reported to last a considerable duration, such as more than a year. Most trials did not clearly specify if participant recruitment was blind to the treatment allocation, and we assumed it was not blind unless specifically mentioned. Conversely, for those trials without any active patient recruitment, we assumed any knowledge of the intervention would not influence selection of identification of participants for inclusion, even though in practice these biases can arise in cluster trials without direct recruitment. We also made an arbitrary decision that a deviation from the intended intervention had occurred when more than 10% of the participants were reported not to have received their intended intervention condition, or that the authors had reported significant concerns around deviations. The issue of deviation of intended treatments is nuanced for pragmatic trials where the objective is to evaluate the effect of the offer of treatment not necessarily the effect of adherence to the treatment – meaning that this lack of adherence might not be important from a pragmatic perspective.

### 4.3 Research in context

Knowledge of treatment condition at the time of patient recruitment is known to be a risk factor for differential identification and recruitment of participants across treatment arms [1,14,16,37], unless recruitment and identification are conducted by someone blind to the treatment allocation or the inclusion criteria are broad [2,11,14]. Methodological reviews have identified that many cluster trials are at risk of these identification and recruitment biases because they recruit participants with knowledge of allocated treatment and this often manifests in baseline imbalances [1,2,28]. These assessments of risk have taken varying forms and it is difficult to compare across reviews. For example, in a review of recent randomized trials, cluster trials were reported to be more likely to have a significant baseline imbalance on age, whereas individually randomized trials were not [1]. Others have assessed about 40% of cluster trials to be at risk of these types of biases [2,7,28]; and sometimes this has been reported to be somewhat lower despite including many trials with post randomization recruitment [10,13]. Thus, the prevalence of risks of bias due to identification and recruitment reported here is higher than in previous reviews. This is likely explained by the fact that we focused on cluster-randomized trials of individual-level interventions, whereas other reviews have included cluster-level interventions where patient recruitment is less common or may more likely to occur prior to randomization.

We also identified that many trials did not report using an allocation method that was clearly concealed. This information was assessed on the basis of whether the randomization was conducted by someone independent, how the randomization was implemented and whether the clusters were all recruited before randomization. This finding is consistent with findings in individual randomized trials which have also been identified at risk of bias due to implementation of the randomization process [21]. We also identified evidence of lack of clear specification of the primary outcome, primary assessment time and primary analysis method, again similar to that identified in individual randomized trials [29]. Both these apparent risks might represent real threats of bias, or they might represent lack of good reporting practices. Whist we did not directly assess quality of reporting, despite the existence of specific reporting guidelines for cluster trials [5], we identified many elements were not well reported. However, lack of awareness of reporting may reflect a lack of awareness around conduct too. Timeline diagrams provide one method of improving reporting of the elements around timing and blinding status of identification and recruitment of participants [4].

Finally, we identified that the most common reasons for adopting cluster randomization were due to either a concern over contamination or for practical reasons; and this echoes what others have found [30]. In a comparison between a novel treatment and usual care any bias due to contamination will attenuate the true treatment effect [18,26,35]. Yet, in the very specific setting of cluster randomized trials of individual-level interventions with post randomization recruitment without blinding, we have identified a high risk of bias due to the differential recruitment across treatment arms. Individually randomized trials, by their nature of not having to recruit post randomization, would not be at risk of this bias. Biases due to identification and recruitment bias operate in an unpredictable direction. Thus, concerns over contamination is unlikely to be an acceptable justification for using cluster randomization in most evaluations of individual-level interventions with unblinded recruitment. Selecting a cluster randomized trial with knowledge that it will be at high risk of bias and without taking steps to mitigate these risks should be considered a poor use of resource at best and at worst unethical [3]. On the other hand, where interest lies in total effects of individual-level interventions (both direct and indirect benefits), so when contamination a positive feature of implementation, then cluster randomization might be the only design of choice [20].

### 4.4 Recommendations

1. Due to the risks of identification and recruitment bias, opting for a cluster design when individual randomization would be feasible needs a strong justification. Concerns around contamination are unlikely to be acceptable justifications; although estimation of indirect effects might be.
2. When cluster randomization is adopted, we recommend that authors provide a clear justification for the choice of cluster randomization and clearly outline strategies to mitigate increased risks of bias. This should include identification and recruitment by someone blind to the treatment allocation and minimal or objective individual-level eligibility criteria.
3. Other good conduct procedures which are routinely implemented in individually randomized trials should be followed. These include implementation of the randomization using an accepted method of allocation concealment, for example, by using an independent statistician to generate the allocation sequence; blind outcome assessment when outcomes are subjective; and clear pre-specification (in a protocol or trial registration site) of the primary outcome including primary assessment time and method of primary analysis.
4. All these aspects should be clearly reported as per CONSORT guidelines. To ensure particular clarity around identification and recruitment, authors should also provide a timeline-cluster diagram.

## Acknowledgments

Acknowledgments are given to Stuart Nicholls (SN, snicholls@ohri.ca) Kelly Carroll (KC, kecarroll@ohri.ca) and Austin R Horn (ARH, ahorn5@uwo.ca) for undertaking search to identify studies; and to Caroline Kristunas (c.a.kristunas@bham.ac.uk) and James Martin (j.martin@bham.ac.uk) for helping with the data abstraction.

## Author contributions

KH led the development of the project and wrote the first draft of the paper. MT led the search process and led the identification of studies for inclusion. CE designed and developed the data abstraction tools and conducted the statistical analysis. MT, SE and JT provided important oversight to the project. All authors helped develop the data abstraction tools, provided critical insight, contributed to the data abstraction exercise, and commented on the draft paper.

## Appendix Supplementary materials

Image, application 1
