# Supplementary material for: Cluster randomized trials of individual-level interventions were at high risk of bias
Source: J Clin Epidemiol. 2021 Oct;138:49–59. doi: 10.1016/j.jclinepi.2021.06.021 (PMC8592576; doi:10.1016/j.jclinepi.2021.06.021)
Supplement: Supplementary file 1 [file mmc1.docx]

**Supplementary Table 1a: Breakdown of risk of bias assessment for Domain 1a by signalling questions**

| **Domain** | **Risk** | **Risk of bias**  **n (%)** |
| --- | --- | --- |
| 1a - Bias arising from the randomization process | Low risk | 12(30) |
|  | Some concerns | 7(17.5) |
|  | High risk | 21(52.5) |
| **Signalling questions** |  |  |
| 1a.1 Was the allocation sequence random? | Yes | 40(100) |
|  | No | 0(0) |
|  | No information | 0(0) |
| 1a.2 Was the allocation sequence concealed  until clusters were enrolled and assigned  to interventions? | Yes | 21(52.5) |
|  | No | 13(32.5) |
|  | No information | 6(15) |
| 1a.3 Were there baseline imbalances that suggest a problem with the randomization process? | Yes | 3(7.5) |
|  | No | 7(17.5) |
|  | No information | 30(75) |

*Note question 1a.2 was in an earlier version of RoB2 (cluster adaption) “Is it likely that the allocation sequence was subverted” although the elaboration / explanation around this question remained the same.

**Supplementary Table 1b: Breakdown of risk of bias assessment for Domain 1b by signalling questions**

| **Domain** | **Risk** | **Risk of bias**  **n (%)** |
| --- | --- | --- |
| 1b - Bias arising from the timing of identification and recruitment of individual participants in relation to timing of randomization | Low risk | 9(22.5) |
|  | Some concerns | 4(10) |
|  | High risk | 27(67.5) |
| **Signalling questions** |  |  |
| 1b.1 Were all the individual participants identified before randomization of clusters (and if the trial specifically recruited patients were they all recruited before randomization of clusters)? | Yes | 3(7.5) |
|  | No | 35(87.5) |
|  | No information | 2(5) |
| 1b.2 If N/PN/NI to 1b.1: Is it likely that selection of individual participants was affected by knowledge of the intervention? | Yes | 27(67.5) |
|  | No | 9(22.5) |
|  | No information | 1(2.5) |
|  | Not applicable | 3(7.5) |
| 1b.3 Were there baseline imbalances that suggest differential identification or recruitment of individual participants between arms? | Yes | 15(37.5) |
|  | No | 18(45) |
|  | No information | 7(17.5) |

**Supplementary Table 2: Breakdown of risk of bias assessment for Domain 2 by signalling questions**

| **Domain** | **Risk** | **Risk of bias**  **n (%)** |
| --- | --- | --- |
| 2 - Bias due to deviations from intended interventions | Low risk | 34(85) |
|  | Some concerns | 0(0) |
|  | High risk | 6(15) |
| **Signalling questions** |  |  |
| 2.1a Were participants aware that they were in a trial? | Yes | 27(67.5) |
|  | No | 11(27.5) |
|  | No information | 2(5) |
| 2.1b If Y/PY/NI to 2.1a: Were participants aware of their assigned intervention during the trial? | Yes | 20(50) |
|  | No | 8(20) |
|  | No information | 1(2.5) |
|  | Not applicable | 11(27.5) |
| 2.2. Were carers and trial personnel aware of participants' assigned intervention during the trial? | Yes | 34(85) |
|  | No | 6(15) |
|  | No information | 0(0) |
| 2.3. If Y/PY/NI to 2.1 or 2.2: Were there deviations from the intended intervention beyond what would be expected in usual practice? | Yes | 8(20) |
|  | No | 32(80) |
|  | No information | 0(0) |
|  | Not applicable | 0(0) |
| 2.4. If Y/PY to 2.3: Were these deviations from intended intervention unbalanced between groups and likely to have affected the outcome? | Yes | 6(15) |
|  | No | 2(5) |
|  | No information | 0(0) |
|  | Not applicable | 32(80) |
| 2.5a Were any clusters analysed in a group different from the one to which they were assigned? (This includes stepped wedge trials which delayed their start dates) | Yes | 0(0) |
|  | No | 40(100) |
|  | No information | 0(0) |
| 2.5b Were any participants analysed in a group different from the one to which their original cluster was randomized?  (This will include cluster contamination in any unidirectional cross over trials) | Yes | 0(0) |
|  | No | 40(100) |
|  | No information | 0(0) |
| 2.6 If Y/PY/NI to 2.5: Was there potential for a substantial impact (on the estimated effect of intervention) of analysing participants in the wrong group? | Yes | 0(0) |
|  | No | 0(0) |
|  | No information | 0(0) |
|  | Not applicable | 40(100) |

Participants were assumed to be aware they were in a trial if the trial reported any mention of informed consent method; If the participants were unblinded to the treatment and control conditions it was assumed they knew of the interventions being compared and their own treatment group; Deviations from intended interventions were assessed as present when more than 10% of participants did not receive the intended intervention condition, or the authors presented other concerns over deviations (supplementary Table 3).

**Supplementary Table 3: Breakdown of risk of bias assessment for Domain 3 by signalling questions**

| **Domain** | **Risk** | **Risk of bias n(%)** |
| --- | --- | --- |
| 3 - Bias due to missing outcome data | Low risk | 33(82.5) |
|  | Some concerns | 5(12.5) |
|  | High risk | 2(5) |
| **Signalling questions** |  |  |
| 3.1a Were outcome data available for all, or nearly all, clusters randomized? ^1^ | Yes | 36(90) |
|  | No | 4(10) |
|  | No information | 0(0) |
| 3.1b Were outcome data available for all, or nearly all, participants within clusters? ^1^ | Yes | 31(77.5) |
|  | No | 9(22.5) |
|  | No information | (0) |
| 3.2 If N/PN/NI to 3.1a or 3.1b: Are the proportions of missing outcome data and reasons for missing outcome data similar across intervention groups? | Yes | 3(7.5) |
|  | No | 4(10) |
|  | No information | 6(15) |
|  | Not applicable | 27(67.5) |
| 3.3 If N/PN/NI to 3.1a or 3.1b: Is there evidence that results were robust to the presence of missing outcome data? | Yes | (0) |
|  | No | 3(23.08) |
|  | No information | 10(76.92) |
|  | Not applicable | 27(67.5) |

^1^ A 90% cut off was used to define nearly all.

**Supplementary Table 4: Breakdown of risk of bias assessment for Domain 4 by signalling questions**

| **Domain** | **Risk** | **Risk of bias**  **n (%)** |
| --- | --- | --- |
| 4 - Bias in measurement of the outcome | Low risk | 31(77.5) |
|  | Some concerns | 0(0) |
|  | High risk | 9(22.5) |
| **Signalling questions** |  |  |
| 4.1a Were outcome assessors aware that a trial was taking place? | Yes | 36(90) |
|  | No | 0(0) |
|  | No information | 4(10) |
| 4.1b If Y/PY/NI to 4.1a: Were outcome assessors aware of the intervention received by study participants? | Yes | 26(65) |
|  | No | 11(27.5) |
|  | No information | 3(7.5) |
|  | Not applicable | 0(0) |
| 4.2 If Y/PY/NI to 4.1b: Was the assessment of the outcome likely to be influenced by knowledge of intervention received? | Yes | 9(22.5) |
|  | No | 20(50) |
|  | No information | 0(0) |
|  | Not applicable | 11(27.5) |

**Supplementary Table 5: Breakdown of risk of bias assessment for Domain 5 including signalling questions**

| **Domain** | **Risk** | **Risk of bias**  **n(%)** |
| --- | --- | --- |
| 5 - Bias in selection of the reported results | Low risk | 18(45) |
|  | Some concerns | 0(0) |
|  | High risk | 22(55) |
| **Signalling questions** |  |  |
| Are the reported outcome data likely to have been selected, on the basis of the results, from … |  |  |
|  |  |  |
|  |  |  |
| 5.1. ... multiple outcome measurements (e.g. scales, definitions, time points) within the outcome domain? | Yes | 14(35) |
|  | No | 26(65) |
|  |  |  |
| Has the primary outcome been pre-specified? | Yes  No | 33(82.5)  7(17.5) |
| For the primary outcome, was an assessment time specified? | Yes  No | 31(77.5)  9(22.5) |
| 5.2 ... multiple analyses of the data? | Yes | 16(40) |
|  | No | 24(60) |
|  |  |  |
| Was it detailed whether the primary outcome results would be reported as adjusted or unadjusted (except for clustering)? | Yes  No | 34(85)  6(15) |
| For the primary outcome, was it detailed that the analysis would account for clustering?* | Yes  No | 35(87.5)  5(12.5) |
| For the primary outcome, Was there pre-specified methods for missing data if there were <90% participant outcome data available? | Yes (and missing data)  Yes (but no missing data)  No (and missing data)  No (but no missing data) | 0(0)  6(15)  25(62.5)  9(22.5) |
| Were there details on what scale the primary outcome results would be given on? | Yes  No | 39(97.5)  1(2.5) |
| If the primary outcome measure was initially a continuous variable but for the purpose of this study it was dichotomised or categorised, was it clear what cut point was going to be used? | Yes  No  Not continuous  Continuous but not categorised  No information | 3(7.5)  0(0)  29(72.5)  7(17.5)  1(2.5) |

The primary outcome was assessed to be pre-specified when it was documented in the protocol paper or trial registrations; or in the methods section of the main trial report. All other assessments also considered documentation in the methods section as pre-specification. *An acceptable allowance for clustering consisted of modelling using mixed models, generalised estimating equations, robust variance estimation of a cluster-level analysis.

**Supplementary Table 6: Supporting documentation for assessment of risk under identification and recruitment bias**

| **Study author and ID** | | **Signalling question** | | | **Overall RoB for section 1b** | **Quotes from main paper/protocol/trial registration and further comments** | | | **Summary statements** | |
| --- | --- | --- | --- | --- | --- | --- | --- | --- | --- | --- |
| Study number | Study author | Signalling question 1b.1:  Were all the individual participants identified before randomization of clusters (and if the trial specifically recruited patients were they all recruited before randomization of clusters)? | Signalling question 1b.2:  If N/PN/NI to 1b.1: Is it likely that selection of individual participants was affected by knowledge of the intervention? | Signalling question 1b.3:  Were there baseline imbalances that suggest differential identification or recruitment of individual participants between arms? | Risk of bias | Participants identified pre or post randomisation?  (Continuous recruitment?) | Active/direct participant recruitment?  (Consent sought?) | Blinding from the intervention status  (Who was blinded?) | Identification and recruitment statement | Baseline imbalance summary |
| 1 | [Sur 2009] | No | Yes | Yes | High | “The vaccines were administered between November 27 and December 31, 2004…All cluster residents were eligible to receive a study vaccine if they were 24 months of age or older, had no reported fever or had an axillary temperature of no more than 37.5°C at the time of administration, and were not pregnant or lactating.” | “All subjects or their guardians provided written informed consent.” | “…the two vaccines were not packaged in an identical fashion.”  “The vaccine manufacturer conducted serologic assays in a blinded manner but played no role in the design of the study, in data analysis, or in the preparation of the manuscript.” | Participants were directly and continuously recruited into the study. Blinding was explicitly mentioned but not with regards to recruiting participants and so assumed that they were not blinded. | There is a suggestion of imbalance, most notably religion. |
| 2 | [de Smet 2009] | No | No | Yes | Some concerns | “Patients admitted to the ICU with an expected  duration of mechanical ventilation of more than  48 hours or an anticipated ICU stay of more than  72 hours were eligible.” | “After reviewing the protocol, the boards  waived the requirement for informed consent” | “Blinding of physicians (or having a third person, who was unaware of the assigned interventions, overseeing inclusion) was deemed impossible.” | Identification of participants was completed at admission to ICU thus continuous identification of participants throughout the study, although consent was waived. | Authors have stated that there are differences at baseline across the treatment groups. The baseline table suggests some differences which concurs with the authors assessment. |
| 3 | [Septimus 2014] | No | No information | No | Some concerns | “Patients admitted to adult ICUs from July 1, 2009, to September 30, 2011.” | Not mentioned. | No mention of blinding to the intervention status. | Identification of participants was completed at admission to ICU, thus continuous recruitment throughout the study. Consent and blinding were not reported and so it is unlikely that they occurred. | The authors state that there is balance at baseline across the intervention and control conditions. The baseline table indeed looks similar across the arms. |
| 4 | [Huang 2013] | No | No | No | Low | “All adult ICUs in a participating hospital were assigned to the same study group.” | “The requirement  for written informed consent was waived” | No mention of blinding to the intervention status in either the main paper or trial registration. | Identification of participants was completed at admission to ICU thus continuous identification of participants throughout the study, although consent was waived. Blinding was not reported and so unlikely that this occurred. | The characteristics appear balanced across the arms. |
| 5 | [Jairath 2015] | No | Yes | Yes | High | “Between Sept 3, 2012, and March 1, 2013, we enrolled 936 patients across six hospitals” | “We sought  written informed consent from individual patients or  their representatives” | “All clinicians, patients, and outcome assessors were unmasked to treatment allocation.” | Participants were directly and continuously recruited throughout the study. The intervention was unblinded. | Authors acknowledge that there are baseline imbalances. The baseline table exhibits some, for example, hypertension. |
| 6 | [Juthani-Mehta 2015] | No | Yes | No | High | “After enrolment [of prevalent participants],  the randomization status of the home was revealed”  “After prevalent participant  enrolment was complete, subsequent screening continued to identify newly admitted or newly eligible residents at each home.” | “…participants or their surrogates provided written consent.” | “Blinded study personnel performed screening assessments and approached eligible residents (or designated surrogates) for consent (i.e., “prevalent participants”).”  “After prevalent participant  enrolment was complete, subsequent screening continued to identify newly admitted or newly eligible residents at each home.”  50/50 split | Participants were directly and continuously recruited throughout the study. Although the study personnel recruiting initial participants were blinded, randomisation status was subsequently revealed during the recruitment of many other participants. | Baseline characteristics and numbers in each arm appear similar. |
| 7 | [Ononge 2015] | No | Yes | No | High | “The study participants were recruited from six health facilities  in Mpigi district, Uganda, between February 2013 and March 2014.” | “After information and counselling, eligible women provided written informed consent…” | “Because of the nature of the intervention, it was not possible to blind the intervention to the care-givers, research team or study participants.” | Participants were directly and continuously recruited throughout the study unblinded to intervention condition. | Baseline characteristics seemed to be balanced in general. |
| 8 | [Climo 2013] | No | No | No information | Low | “…involving patients hospitalized in six ICUs  or bone marrow transplantation units between  August 2007 and February 2009.” | “Waiver of written informed consent was obtained at each institution, owing to the minimal-risk nature of the study.” | “We conducted a multicenter, cluster-randomized, nonblinded crossover trial…” | Participants were continuously identified for eligibility although they were not directly recruited into the study. The study as unblinded. | Baseline characteristics at the participant level were not recorded. |
| 9 | [Stanton 2013] | No | Yes | Yes | High | “We enrolled 689 and 897 women, respectively, into oxytocin and control arms of the trial from April 2011 to November 2012.” | “…field workers sought written initial informed consent for the trial.”  “Enrolled women are those who provided final consent…” | “Limitations include an unblinded trial…” | Participants were directly and continuously recruited throughout the study without blinding. | There are multiple baseline characteristics which appear unbalanced across the arms. |
| 10 | [Churchyard 2014] | No | Yes | No | High | “In intervention  clusters, miners were recruited during the intervention  enrolment period, with the duration of the enrolment period varying according to cluster size and continuing until all miners had the opportunity to enrol. The subsequent 9-month intervention follow-up period allowed all participants to complete 9 months of isoniazid therapy; miners who joined intervention workforces during this period were offered enrolment.” | “Miners who participated in the intervention  and prevalence surveys provided written or  witnessed oral informed consent” | No explicit mention of blinding in the main paper. In the trial registration (ISRCTN) it is stated that this is a non-blinded trial. | Participants were directly and continuously recruited throughout the study without being blinded. | The baseline characteristics appear similar across the arms. |
| 11 | [Siebers 2009] | No | Yes | Yes | High | “Recruitment started in April 2003 and concluded in July 2006 after the enrolment of 89 960 women.” | “Informed refusal was offered with an information folder.” | “To prevent selective assessment  bias, study personnel—gynecologists, pathologists, cytotechnologists, and others—involved in the follow-up and review of histology and cytology were blinded to the cytology screening system used.” | Participants were continuously recruited throughout the study and direct recruitment occurred by way of ‘informed refusal’. Blinding was explicitly mentioned for follow up assessors was stated, however there was no mention of blinding of those that were recruiting participants, so presumed they were not blinded. | Multiple characteristics appear to be imbalanced across the arms. |
| 12 | [Labbe 2012] | No | No | Yes | Some concerns | “During a  nine-month period (from March 2001 in Benin and from September  2001 in Ghana), FSWs from these clusters presenting to dedicated clinics were invited to participate.” | “Informed and signed consent was obtained from all participants.” | “…double blind, placebo-controlled trial…” | Recruitment of participants occurred after the randomisation of clusters. This was a placebo-controlled trial. | The baseline characteristics table suggests that there is imbalance across arms for multiple characteristics. |
| 13 | [Palmu 2013] | No | No | No | Low | “Participants were enrolled  and were given vaccinations in local well-baby clinics  (N=651) by the nurses (N>2200) who are responsible for routine health follow-up and immunisations.” | “Well-baby-clinic nurses (and physicians when needed) gave oral information and obtained the written informed consent from a parent or legal guardian.” | “Each well-baby clinic was provided with two boxes of identical vials of study vaccine: one for children enrolled before 12 months (PHiD-CV10 or hepatitis B virus vaccine) and another for children enrolled at an older age (PHiD-CV10 or hepatitis A virus vaccine).”  “The number or the borders of the clusters were not revealed to participants or nurses and physicians at well-baby clinics. The clinicians diagnosing and treating invasive pneumococcal disease were not otherwise involved in the trial conduct. The investigators were masked to the participant and cluster details of the cases of invasive pneumococcal disease occurring during the study.” | Participants were directly and continuously recruited throughout the study. Blinding to the invention occurred in way of a sham control and masking of all study personnel. | The baseline characteristics table suggests that there is similarities and balance across arms. |
| 14 | [Kalra 2015] | No | Yes | No | High | “We randomly assigned the units (1:1) by computer to give either prophylactic antibiotics for 7 days plus standard stroke unit care or standard stroke unit care only to patients clustered in the units within 48 h of stroke onset.” | “Patients or their next of kin provided written informed  consent or assent, respectively” | “Patients, research staff obtaining data, and statisticians undertaking analyses of the outcome data were unaware of stroke unit allocation.” | Participants were directly and continuously recruited throughout the study. Blinding was explicitly mentioned for follow up assessors was stated, however there was no mention of blinding of those that were recruiting participants, so presumed they were not blinded. | The baseline characteristics table suggests that there is similarities and balance across arms. |
| 15 | [Tielsch 2007] | No | No | No | Low | “Pregnancies in the study area were identified by the  WDs, who went door to door on a monthly basis. At 6 months’ gestation, women were recruited for participation.” | “Informed consent was obtained at the community, household, and individual levels. Community consent was obtained during meetings with community leaders.  In addition, verbal consent was obtained at the household  level from the parents of enrolled infants” | “The allocation codes were kept at  Proctor and Gamble, and investigators and all study staff  were masked to the treatment assignment” | Participants were directly and continuously recruited throughout the study. Blinding to the invention occurred by masking of all study personnel through the use of a placebo. | The baseline characteristics table suggests that there is similarities and balance across arms. |
| 16 | [Gebre 2012] | No | Yes | No information | High | “Enrolment and  recruitment began in June, 2006, and continued until the  final treatment and follow-up in November, 2009” | “Informed consent from the parent or guardian was  obtained (owing to the high rates of illiteracy in the region,  fieldworkers read out the consent form and obtained verbal  consent from the participants), as well as verbal agreement from children who were at least 7 years of age.” | “Treatment allocation and time point were masked from laboratory workers; however, they were aware of the relative time of the study as it progressed over the full 42 months. Treatment assignment was not masked from state team members.” | Participants were directly and continuously recruited throughout the study. Blinding to the intervention allocation was stated for outcome assessors but the remaining study members were ‘not masked’ to ‘treatment assignment’. | No information given at the individual participant level. |
| 17 | [Hejblum 2009] | No | Yes | Yes | High | “The first patient was enrolled on Dec 21, 2006, and the  last was discharged on Aug 22, 2007;”  “We planned to recruit the same number of patients from every intensive care unit to better account for variability between units. Consequently, the duration of the study varied between units dependent on baseline recruitment and case-mix.” | “According to the French regulation on clinical research using standard care procedures, 21 informed consent was unnecessary, and instead patients were given information about the study. This  information was posted in the visitor’s waiting room of  every intensive care unit, and included the procedure  by which the patients could access their data and confirmed that patients could refuse to participate.” | “The study was open-label with respect to allocation concealment and masking of intervention for practical reasons.” | Participants were continuously recruited throughout the study and direct recruitment occurred. This was an ‘open-label’ study and therefore suggest that recruiting personnel were aware of treatment allocation. | There is a suggestion of imbalance across the arms within the baseline table, most notably 14 percentage point difference for a particular characteristic. |
| 18 | [Coulibaly 2013] | No | Yes | No | High | “Patients were identified after randomization since the whole village was allocated to the same treatment.” | “Subsequently, potential subjects in the study provided their own written consent to information provided on audiotape in Bambara (the local language). Parents or guardians provided written consent for all children participating in the study.” | “The study was an open label…” | Participants ‘were identified after randomisation’ and directly recruited. This was an ‘open-label’ study and therefore suggest that recruiting personnel were aware of treatment allocation. | On the whole the baseline characteristics suggests balance across arms. |
| 19 | [Moore 2011] | No information | Yes | Yes | High | Not enough information – long term care facilities and so possibly recruitment before randomisation, but unable to determine. | “The researcher then visited the patients, having  allowed them time to absorb the information (at least  24 hours later), to obtain consent” | “A pragmatic, multi-centre, open label, prospective, cluster-randomised controlled trial…” | Participants were directly recruited to the study. Although it was unclear if participants were before randomisation. This was an open-label study and therefore suggest that recruiting personnel were aware of treatment allocation. | The baseline characteristics that were reported suggest that there was notable imbalance between across the arms. |
| 20 | [Soofi 2012] | No | Yes | No | high | “All newborn babies delivered participating TBAs were eligible for enrolment in the study.” | “Liveborn infants delivered by participating TBAs who received birth kits were eligible for enrolment in the study.” | “Implementation and data collection teams were masked to allocation.”  Trial registration states open label. | Participants were continuously recruited throughout the study. Although consent was not explicitly stated, there is a suggestion that direct recruitment occurred as terminology such as ‘enrolment’ was used. ‘Implementation and data collection teams were masked to allocation’, however not explicitly stated for recruiters, thus assumed they were not blinded. | The baseline characteristics appear fairly balanced across arms. |
| 21 | [Booy 2012] | No | Yes | Yes | High | “The study period 30 June  2006 to 23 December 2008 included three southern hemisphere winters. During that time, influenza was confirmed as the  cause of nine of the 23 ILI outbreaks that occurred amongst the 16 ACFs” | “…provided individual written consent was obtained from the subject or the subject’s guardian/legal representative (when dementia or other incapacity was present).” | “ACF and study staff were not blinded once random allocation was generated.” | Participants were directly and continuously recruited throughout the study. ‘Study staff were not blinded once random allocation was generated.’ | There was an indication of imbalance across the arms and the types of participants included in the study. |
| 22 | [Freitag 2007] | No | Yes | No | High | “At Visit 1 [post randomisation of clusters], inclusion and exclusion criteria were reviewed to determine patients’ eligibility.  Patients were provided with written informed consent.” | “Patients were required to sign a written  informed consent regarding the study and a Health Insurance  Portability and Accountability Act (HIPAA) consent explaining the use of the information collected during this study.” | “open-label” | Participants were directly and continuously recruited throughout the study. This was an ‘open-label’ study and therefore suggest that recruiting personnel were aware of treatment allocation. | The baseline characteristics look balanced across arms. |
| 23 | [Cox 2014] | No | No | No | Low | “Participants enrolled during each designated Xpert week had Xpert requested on the routine laboratory request forms.” | “Individual informed consent at study inclusion was waived for this study based on the pragmatic nature of the trial.” | “Clinic staff and investigators  were unblinded to the intervention” | Participants were directly and continuously recruited throughout the study. ‘Clinic staff and investigators were unblinded to the intervention’. There was no explicit mention of recruiters but assumed that they were also ‘unblinded’. | The baseline characteristics look balanced across arms. |
| 24 | [Stiell 2011] | No | No | No information | Low | “All episodes of cardiac arrest in a cluster were randomly assigned to one CPR strategy; after a set period of time, ranging from 3 to 12 months, all episodes in that cluster were then assigned to the other strategy. All the clusters were assigned to cross over to the other strategy one or more times during the study at fixed intervals; we estimated that approximately 100 patients would be included during each interval.”  Recruitment was post randomisation | “Patients were not required to provide informed consent;” | “Patients in the early-analysis group were assigned to receive 30 to 60 seconds of  EMS-administered CPR and those in the later-analysis group were assigned to receive  180 seconds of CPR, before the initial electrocardiographic analysis”  “single-blinded cluster randomized crossover controlled trial” [protocol paper] | Participants were recruited post randomisation and were not directly recruited. The protocol paper states that this was a ‘single blinded’ study. The nature of the intervention implies that the patient did not know of the intervention at the time it was given. Thus with no explicit mention of recruiters it was assumed they were not blinded to the treatment allocation. | It was unclear if there was imbalance across arms due to the reporting of the baseline characteristics. |
| 25 | [Reynolds 2010] | No | Yes | No | High | “The first interview took place at the time of recruitment into the study after their ANC visit. This interview contained information about how to contact the mother for the follow up interview, her estimated date of delivery, … “ | “…and gave signed informed consent to participate were enrolled in the study.” | Not mentioned | Participants appear to be directly and continuously recruited throughout the study. Although blinding was not explicitly mentioned, the nature of the intervention did not lend itself to be blinded to. | There is no apparent imbalance across the arms for baseline characteristics. |
| 26 | [Karkouti 2016] | No | No | Yes | Some concerns | “The trial included all patients who underwent cardiac surgery with CPB during the study period.” | “…and all participating hospitals, all of which waived the need for informed consent from individual patients.” | “Within 1 month before algorithm initiation, clinical and research staff received on-site training for performing the assays, implementing the algorithm, and collecting the data.” | Participants were continuously identified throughout the study, although. consent was waived. Although not explicit it is unlikely that those identifying eligible participants were blinded due to the nature of the intervention. | There is a suggestion of imbalance within the baseline characteristics across arms. |
| 27 | [Miller 2013] | No | Yes | No | High | “…enrollment ended May 2012, approximately 30 months post-randomization.” | “Written informed consent (signature from literate participants and thumb print from those who could not sign) was obtained from all study participants who were conscious and able to give consent.”  “Participants were consented when they became eligible, if they were conscious and able to give consent. All ethics committees  approved waiver of consent for unconscious women; consent for  an unconscious women was either obtained from a relative and/or the patient after she regained consciousness.” | “We chose a cluster randomized design because it is impossible to blind providers to the intervention or to develop a placebo garment.”  “Because the NASG is visible, blinding of participants and clinicians/data collectors was impossible; the UCSF research team was blind to outcomes.” | Participants were directly and continuously recruited throughout the study. Blinding ‘of the participants and clinicians/data collectors was impossible’, however blinding of recruiters was not explicitly mentioned but assumed that they were not blinded to the treatment allocation. | There seems to be balance across the arms for baseline characteristics. |
| 28 | [West 2013] | No information | Yes | No information | High | No information | “Written informed consent was obtained by parents on behalf of all child participants.” | “The survey teams who assessed trachoma were masked to the allocation of the communities in each arm … and these were processed for infection masked to intervention. The community residents who participated in the survey were not told the results…Thus, the infection outcomes were double masked.” | It is unclear if participants were directly recruited into the study post randomisation. ‘Masking’ occurred for the outcome assessors but not explicit mention of recruiters being masked therefore it was assumed that they were not blinded to the treatment allocation. | There are no individual participant characteristics reported within the paper to assess imbalance across arms. |
| 29 | [Chinbuah 2012] | No | Yes | Yes | High | “Children as they turned 2 months and children 2–59 months of age who moved into the district during the study were included.” | “Written individual informed consent was obtained from all caregivers in the first year to determine their willingness to participate in the entire trial, but written informed consent was also obtained from parents or guardians for all subsequent surveys or interview(s).” | “..open trial,…” | Participants were directly and continuously recruited throughout the study. This study was also an ‘open trial’. | There is a suggestion of imbalance across arms for multiple baseline characteristics. |
| 30 | [Metsvaht 2010] | No | Yes | No information | High | “During the study period all neonates admitted within 72 h  of life, needing early empiric antibiotic treatment for EOS  or risk factors of infection according to the CDC criteria  (e.g. maternal chorionamnionitis and ⁄ or maternal risk factors  of infection and ⁄ or preterm labour in <35 weeks of gestation) and not transferred within the following 24 h, were eligible.” | “In the second phase (from 21 March 2007 to 30 November 2007), after half of the patients were recruited, beta-lactam antibiotics were switched.” | “..open label..” | Participants were continuously recruited throughout the study. There was no explicit mention on if consent was taken, however words such as ‘recruited’ has been used implying direct recruitment. This was also a ‘open label’ study. | It was unclear if there was imbalance across arms due to the reporting of the baseline characteristics. |
| 31 | [Gater 2010] | Yes | N/A | No | Low | “Written informed consent was sought at screening and then again before randomisation.”  “In a two-phase procedure, the Self-Reporting Questionnaire  (SRQ) was administered to identify probable cases. … Clinical Interview to confirm a diagnosis of depression.” | “Written informed consent was sought at screening and then again before randomisation.” | “Three British Pakistani postgraduate researchers received initial  2-week training on the use of assessment schedules and culturally sensitive research methods focusing on recruitment, consent and trial procedures. Researchers independent of those providing the intervention and masked to the intervention allocation and adherence undertook outcome assessments.” | Participants were directly recruited into the study before randomisation took place. | Across the arms the baseline characteristics appear similar. |
| 32 | [Awasthi 2013] | No | Yes | No information | High | “A total of about one million  preschool children will therefore be in the study at any one time; as the study will continue  for about 5 years, with children entering it as they reach their first birthday and leaving as they reach their sixth, a total of about 2 million preschool children will be in it at one time or another, and a total of about 5 million person-years of experience will accumulate.” [study protocol paper] | “Villagers decided after public discussion whether the village would cooperate; if it did, no caregiver then chose to refuse consent. Only one selected village refused; an adjacent village replaced it.  After marking consent, caregivers were asked whether  the previous mass-treatment had been received” | “No placebos were used; the control was open.” [Trial registration database] | Participants were directly and continuously recruited throughout the study. The trial registration for this study stated that there was no ‘masking’ and it was ‘open label’, with the main paper stating that ‘no placebos were used; the control was open’. | There are no individual participant characteristics reported within the paper to assess imbalance across arms. |
| 33 | [Juan-Giner 2014] | No | Yes | No | High | “On the day of inclusion into the study, … the vaccinator adminis-tered the 1st dose of TT vaccine…” | “The head of each participating village provided permission for their village to participate and written informed consent was obtained before enrollment from all participants.” | “While vaccinators and health personnel conducting the study were aware of allocation group, village heads, participants and laboratory personnel analyzing samples were blinded to the allocation.” | Participants were directly and continuously recruited throughout the study. ‘Vaccinators and health personnel con-ducting the study were aware of allocation group’, with others (not including recruiter) being blinded to the treatment. | The baseline characteristics are appear similar across arms. |
| 34 | [Roca 2011] | No | Yes | No | High | “All residents of selected 21 Gambian villages are eligible including those born during the study.” “In the lifetime of the study, new settlers in the villages will be identified and recruited for the study. The same vaccination schedule will apply.” [Protocol paper] | “Study participants gave individual informed consent; parental consent was obtained for children up to 16 y of age.” | “Blinding included vaccine recipients and laboratory personnel. Study nurses were aware of the nature of the vaccines given but played no other part in the conduct of the trial.” – from the main paper.  “This study is a single-blind placebo-controlled community randomized trial” [Protocol paper] | Participants were directly and continuously recruited throughout the study. Blinding only occurred for the participants and laboratory staff. There was no explicit mention of recruiters being blind to the treatment allocation and so assumed that they were not. | The baseline characteristics are appear similar across arms. |
| 35 | [Neuzil 2011] | Yes | N/A | Yes | Low | “Healthy adolescent girls aged 11 through 13 years in grades 6 or 7 were enrolled in the study.”  The figure presented in the main paper gives details on recruitment of participants which appears to occur before randomisation. | “Written informed consent was obtained from all parents and written assent was obtained from all participants.” | “..open-label..” | Participants were directly recruited into the study before randomisation as consent was taken before this occurred. | The baseline characteristics show some suggestion that there are imbalances across arms. |
| 36 | [Tine 2011] | Yes | N/A | No | Low | “meetings were held in the villages to explain the study purpose and answer to the population’s questions. Consent was obtained from the community leaders as well as parents or children’s guardians.”  The flow diagram also reflects this ordering. | “Consent was obtained from the community leaders as well as parents or children’s guardians.” | Not mentioned. | Participants were directly recruited into the study. Consent was taken at the time that clusters were recruited into the study, which appeared to be before randomisation was conducted. | The baseline characteristics appear similar across arms. |
| 37 | [Long 2016] | No | Yes | Yes | High | “Participants were vaccinated in the morning or afternoon between 2011 and 2013.” | “Intervention participants were invited to take part in the study by a letter sent from their GP surgery on behalf of the research sponsor (University of Birmingham (UB)) and they returned the signed written informed consent form to the research team at UB.” | “This was a non-blinded cluster-randomised trial.” | Participants were directly and continuously recruited throughout the study. This was also a ‘non-blinded’ study. | The baseline characteristics show some suggestion that there is imbalances across arms. |
| 38 | [Pearce 2013] | No | Yes | No information | High | “and unblinded IPTi implementation started in all 62 vaccinating health facilities in intervention divisions in April 2005. Tablets of SP were offered to children  attending facilities for routine vaccinations with diphtheria–  tetanus toxoid–acellular pertussis, hepatitis B virus, and oral  poliovirus vaccine, at approximately 2 and 3 months of age,  and a third dose was offered when attending for measles vaccination, at about 9 months of age.” | “During field work, information sheets in Swahili were provided, and written informed consent was sought from the heads of all households.” | “Molecular and HPLC laboratory analyses were conducted  with laboratory workers blind to whether samples came from  IPTi or comparison areas.”  “The districts are subdivided into 24 administrative areas, called divisions. IPTi  was randomly assigned to 12 divisions [28], leaving 12 divisions as controls, and unblinded IPTi implementation started in all 62 vaccinating health facilities in intervention divisions in April 2005.” | Participants were directly and continuously recruited throughout the study. This study was ‘unblinded’. Explicit mention about blinding of recruiters was not stated but presumed did not occur. | There are no individual participant characteristics reported within the paper to assess imbalance across arms. |
| 39 | [Bari 2011] | No | Yes | Yes | High | “LHWs were trained to screen every child presenting to them with cough and difficulty breathing for enrolment.” | “Children’s legal guardians provided written  informed consent” | “Participants, carers, and assessors were not masked to treatment assignment.” | Participants were directly and continuously recruited throughout the study. Explicit mention about blinding of recruiters was not stated but presumed did not occur. | There is some imbalance across the arms within the baseline characteristics. |
| 40 | [Postma 2015] | No | Yes | Yes | High | “Emergency department registries were screened  daily for eligible patients by research nurses or  physicians”  Continuous recruitment | “Obtaining informed consent before intervention was deemed unnecessary, because patients did not undergo randomization individually, and all the antibiotics we studied are used in current practice. Written informed consent obtained within 72 hours after admission was required for data collection.” | “As optimal treatment may require protocol deviation, blinding for treatment is not feasible.” [Protocol] | Participants were continuously recruited throughout the study. Whislt no consent was taken for the intervention, participants were recruited as consent was taken post treatment administration for data collection purposes. As stated in the protocol ‘blinding for treatment is not feasible’. | There is some imbalance across the arms within the baseline characteristics. |

**Supplementary Table 7: Description of supporting justifications where there was assessed to be evidence that the intervention was not delivered as expected**

| **Paper ID** | **Author** | **Supporting quote to justify that the intervention was not delivered as expected** |
| --- | --- | --- |
| 8 | [Climo 2013] | “Sage Products initiated a nationwide recall of the 2% chlorhexidine–impregnated washcloths [intervention condition], because of Burkholderia cepacia contamination of some product lots. Units using the chlorhexidine product at the time of the recall were switched to the nonantimicrobial washcloths [control condition], and the institutional review boards were immediately notified.” |
| 10 | [Churchyard 2014] | “Participation in the intervention and retention in the study were variable across clusters. …In other mines, intervention enrolment took longer, and retention was lower than desirable. Thus, the proportion of miners taking isoniazid simultaneously was suboptimal.” |
| 21 | [Booy 2012] | “In T&P facilities, we were required to obtain consent in all eligible residents in order to deliver the prophylactic arm of the trial. Despite attempts to consent all residents prior to outbreaks, the turnover of residents and huge workload required, meant that we were only able to consent a minority prior to an outbreak” (Note objective was to evaluate effect of treating entire cluster: “… the use of oseltamivir to treat patients in an outbreak may result in herd-protective effects beyond an individual effect.” |
| 23 | [Cox 2014] | “these benefits were seen despite inadequate application of the intervention”  “Overall, we feel the problems with the application of the intervention were predominately because of the crossing over of the intervention using a weekly randomisation schedule…” |
| 26 | [Karkouti 2016] | “…we did not allow for a transition period between the 2 phases of the study, and the last hospitals randomly assigned had only a 1-month exposure to the intervention. Thus, clinicians may not have had adequate time to become fully proficient with the POC assays and transfusion algorithm [intervention condition], which may have caused our trial to underestimate the treatment effect.” |
| 38 | [Pearce 2013] | “First-line treatment was switched from SP to artemether-lumefantrine before the final survey….Between 2004 and 2006, while SP was the first-line treatment for malaria infections and the drug used for IPTi….In late 2006, the national treatment policy with ALu was rolled out, and SP use decreased.” [all changes to standard of care] |

**Supplementary Table 8: Agreement between reviewers during the independent data abstraction process**

| **Signalling question** | **Percentage agreement** | **Gwet’s AC**  **(95% CI)** |
| --- | --- | --- |
| **Domain 1a - Bias arising from the randomization process** | **50** | **0.38(0.16,0.59)** |
| 1 Was the allocation sequence random? | 100 | - |
| 2 Is it likely that the allocation sequence was subverted? (Equivalently: was the allocation concealed) | 52 | 0.41(0.19,0.62) |
| 3 Were there baseline imbalances that suggest a problem with the randomization process? | 80 | 0.75(0.57,0.93) |
|  |  |  |
| **Domain 1b - Bias arising from the timing of identification and recruitment of individual participants in relation to timing of randomization** | **62** | **0.53(0.32,0.74)** |
| 1 Were all the individual participants identified before randomization of clusters (and if the trial specifically recruited patients were they all recruited before randomization of clusters)? | 82 | 0.80(0.64,0.95) |
| 2 If N/PN/NI to 1: Is it likely that selection of individual participants was affected by knowledge of the intervention? | 65 | 0.56(0.36,0.76) |
| 3 Were there baseline imbalances that suggest differential identification or recruitment of individual participants between arms? | 62 | 0.53(0.33,0.73) |
|  |  |  |
| **Domain 2 - Bias due to deviations from intended interventions** | **75** | **0.72(0.55,0.89)** |
| 1a Were participants aware that they were in a trial? | 70 | 0.65(0.46,0.83) |
| 1b If Y/PY/NI to 1a: Were participants aware of their assigned intervention during the trial? | 62 | 0.53(0.33,0.74) |
| 2. Were carers and trial personnel aware of participants' assigned intervention during the trial? | 80 | 0.78(0.63,0.93) |
| 3. If Y/PY/NI to 1 or 2: Were there deviations from the intended intervention beyond what would be expected in usual practice? | 82 | 0.75(0.55,0.95) |
| 4. If Y/PY to 3: Were these deviations from intended intervention unbalanced between groups and likely to have affected the outcome? | 80 | 0.78(0.62,0.93) |
| 5a Were any clusters analysed in a group different from the one to which they were assigned? | 87 | 0.87(0.75,0.99) |
| 5b Were any participants analysed in a group different from the one to which their original cluster was randomized? | 95 | 0.95(0.87,1.00) |
| 6 If Y/PY/NI to 5: Was there potential for a substantial impact (on the estimated effect of intervention) of analysing participants in the wrong group? | 90 | 0.90(0.79,1.00) |
|  |  |  |
| **Domain 3 - Bias due to missing outcome data** | **67** | **0.63(0.44,0.82)** |
| 1a Were outcome data available for all, or nearly all, clusters randomized? | 90 | 0.89(0.78,1.00) |
| 1b Were outcome data available for all, or nearly all, participants within clusters? | 80 | 0.77(0.61,0.92) |
| 2 If N/PN/NI to 1a or 1b: Are the proportions of missing outcome data and reasons for missing outcome data similar across intervention groups? | 65 | 0.59(0.40,0.78) |
| 3 If N/PN/NI to 1a or 1b: Is there evidence that results were robust to the presence of missing outcome data? | 70 | 0.64(0.45,0.83) |
|  |  |  |
| **Domain 4 - Bias in measurement of the outcome** | **75** | **0.70(0.52,0.88)** |
| 1a Were outcome assessors aware that a trial was taking place? | 90 | 0.90(0.79,1.00) |
| 1b If Y/PY/NI to 1a: Were outcome assessors aware of the intervention received by study participants? | 80 | 0.77(0.62,0.92) |
| 2 If Y/PY/NI to 1b: Was the assessment of the outcome likely to be influenced by knowledge of intervention received? | 75 | 0.64(0.44,0.85) |
|  |  |  |
| **Domain 5 - Bias in selection of the reported results** | **57** | **0.43(0.20,0.65)** |
| 1 Are the reported outcome data likely to have been selected, on the basis of the results, from multiple outcome measurements (e.g. scales, definitions, time points) within the outcome domain? | 67 | 0.58(0.36,0.79) |
| 2 Are the reported outcome data likely to have been selected, on the basis of the results, from multiple analyses of the data? | 65 | 0.52(0.30,0.74) |
|  |  |  |
| **Overall risk of bias** | **82** | **0.81(0.67,0.95)** |

**Papers included in the Review**

[Freitag 2007] Freitag FG, Finlayson G, Rapoport AM, Elkind AH, Diamond ML, Unger JR, Fisher AC, Armstrong RB, Hulihan JF, Greenberg SJ. Effect of pain intensity and time to administration on responsiveness to almotriptan: results from AXERT 12.5 mg Time Versus Intensity Migraine Study (AIMS). Headache. 2007;47(4):519-30.

[Tielsch 2007] Tielsch JM, Darmstadt GL, Mullany LC, Khatry SK, Katz J, LeClerq SC, Shrestha S, Adhikari R. Impact of newborn skin-cleansing with chlorhexidine on neonatal mortality in southern Nepal: a community-based, cluster-randomized trial. Pediatrics. 2007;119(2):e330-40.

[de Smet 2009] de Smet AMGA, Kluytmans JAJW, Cooper BS, Mascini EM, Benus RFJ, van der Werf TS, van der Hoeven JG, Pickkers P, Bogaers-Hofman D, van der Meer NJM, Bernards AT, Kuijper EJ, Joore JCA, Leverstein-van Hall MA, Bindels AJGH, Jansz AR, Wesselink RMJ, de Jongh BM, Dennesen PJW, van Asselt GJ, te Velde LF, Frenay IHME, Kaasjager K, Bosch FH, van Iterson M, Thijsen SFT, Kluge GH, Pauw W, de Vries JW, Kaan JA, Arends JP, Aarts LPHJ, Sturm PDJ, Harinck HIJ, Voss A, Uijtendaal EV, Blok HEM, Thieme Groen ES, Pouw ME, Kalkman CJ, Bonten MJM. Decontamination of the Digestive Tract and Oropharynx in ICU Patients. New England Journal of Medicine. 2009;360(1):20-31.

[Hejblum 2009] Hejblum G, Chalumeau-Lemoine L, Ioos V, Boelle PY, Salomon L, Simon T, Vibert JF, Guidet B. Comparison of routine and on-demand prescription of chest radiographs in mechanically ventilated adults: a multicentre, cluster-randomised, two-period crossover study. Lancet. 2009;374(9702):1687-93.

[Siebers 2009] Siebers AG, Klinkhamer PJ, Grefte JM, Massuger LF, Vedder JE, Beijers-Broos A, Bulten J, Arbyn M. Comparison of liquid-based cytology with conventional cytology for detection of cervical cancer precursors: a randomized controlled trial. Jama. 2009;302(16):1757-64.

[Sur 2009] Sur D, Ochiai RL, Bhattacharya SK, Ganguly NK, Ali M, Manna B, Dutta S, Donner A, Kanungo S, Park JK, Puri MK, Kim DR, Dutta D, Bhaduri B, Acosta CJ, Clemens JD. A cluster-randomized effectiveness trial of Vi typhoid vaccine in India. N Engl J Med. 2009;361(4):335-44.

[Gater 2010] Gater R, Waheed W, Husain N, Tomenson B, Aseem S, Creed F. Social intervention for British Pakistani women with depression: randomised controlled trial. Br J Psychiatry. 2010;197(3):227-33.

[Metsvaht 2010] Metsvaht T, Ilmoja ML, Parm U, Maipuu L, Merila M, Lutsar I. Comparison of ampicillin plus gentamicin vs. penicillin plus gentamicin in empiric treatment of neonates at risk of early onset sepsis. Acta Paediatr. 2010;99(5):665-72.

[Reynolds 2010] Reynolds HW, Gachuno O, Kayita J, Hays MA, Otterness C, Rakwar J. Cluster randomised trial of the uptake of a take-home infant dose of nevirapine in Kenya. East Afr Med J. 2010;87(7):284-93.

[Bari 2011] Bari A, Sadruddin S, Khan A, Khan I, Khan A, Lehri IA, Macleod WB, Fox MP, Thea DM, Qazi SA. Community case management of severe pneumonia with oral amoxicillin in children aged 2-59 months in Haripur district, Pakistan: a cluster randomised trial. Lancet. 2011;378(9805):1796-803.

[Moore 2011] Moore Z, Cowman S, Conroy RM. A randomised controlled clinical trial of repositioning, using the 30 degrees tilt, for the prevention of pressure ulcers. J Clin Nurs. 2011;20(17-18):2633-44.

[Neuzil 2011] Neuzil KM, Canh DG, Thiem VD, Janmohamed A, Huong VM, Tang Y, Diep NT, Tsu V, LaMontagne DS. Immunogenicity and reactogenicity of alternative schedules of HPV vaccine in Vietnam: a cluster randomized noninferiority trial. Jama. 2011;305(14):1424-31.

[Roca 2011] Roca A, Hill PC, Townend J, Egere U, Antonio M, Bojang A, Akisanya A, Litchfield T, Nsekpong DE, Oluwalana C, Howie SR, Greenwood B, Adegbola RA. Effects of community-wide vaccination with PCV-7 on pneumococcal nasopharyngeal carriage in the Gambia: a cluster-randomized trial. PLoS Med. 2011;8(10):e1001107.

[Stiell 2011] Stiell IG, Nichol G, Leroux BG, Rea TD, Ornato JP, Powell J, Christenson J, Callaway CW, Kudenchuk PJ, Aufderheide TP, Idris AH, Daya MR, Wang HE, Morrison LJ, Davis D, Andrusiek D, Stephens S, Cheskes S, Schmicker RH, Fowler R, Vaillancourt C, Hostler D, Zive D, Pirrallo RG, Vilke GM, Sopko G, Weisfeldt M. Early versus later rhythm analysis in patients with out-of-hospital cardiac arrest. N Engl J Med. 2011;365(9):787-97.

[Tine 2011] Tine RC, Faye B, Ndour CT, Ndiaye JL, Ndiaye M, Bassene C, Magnussen P, Bygbjerg IC, Sylla K, Ndour JD, Gaye O. Impact of combining intermittent preventive treatment with home management of malaria in children less than 10 years in a rural area of Senegal: a cluster randomized trial. Malar J. 2011;10:358.

[Booy 2012] Booy R, Lindley RI, Dwyer DE, Yin JK, Heron LG, Moffatt CR, Chiu CK, Rosewell AE, Dean AS, Dobbins T, Philp DJ, Gao Z, MacIntyre CR. Treating and preventing influenza in aged care facilities: a cluster randomised controlled trial. PLoS One. 2012;7(10):e46509.

[Chinbuah 2012] Chinbuah MA, Kager PA, Abbey M, Gyapong M, Awini E, Nonvignon J, Adjuik M, Aikins M, Pagnoni F, Gyapong JO. Impact of community management of fever (using antimalarials with or without antibiotics) on childhood mortality: a cluster-randomized controlled trial in Ghana. Am J Trop Med Hyg. 2012;87(5 Suppl):11-20.

[Gebre 2012] Gebre T, Ayele B, Zerihun M, Genet A, Stoller NE, Zhou Z, House JI, Yu SN, Ray KJ, Emerson PM, Keenan JD, Porco TC, Lietman TM, Gaynor BD. Comparison of annual versus twice-yearly mass azithromycin treatment for hyperendemic trachoma in Ethiopia: a cluster-randomised trial. Lancet. 2012;379(9811):143-51.

[Labbe 2012] Labbe AC, Pepin J, Khonde N, Dzokoto A, Meda H, Asamoah-Adu C, Mayaud P, Mabey D, Demers E, Alary M. Periodical antibiotic treatment for the control of gonococcal and chlamydial infections among sex workers in Benin and Ghana: a cluster-randomized placebo-controlled trial. Sex Transm Dis. 2012;39(4):253-9.

[Soofi 2012] Soofi S, Cousens S, Imdad A, Bhutto N, Ali N, Bhutta ZA. Topical application of chlorhexidine to neonatal umbilical cords for prevention of omphalitis and neonatal mortality in a rural district of Pakistan: a community-based, cluster-randomised trial. Lancet. 2012;379(9820):1029-36.

[Awasthi 2013] Awasthi S, Peto R, Read S, Richards SM, Pande V, Bundy D. Population deworming every 6 months with albendazole in 1 million pre-school children in North India: DEVTA, a cluster-randomised trial. Lancet. 2013;381(9876):1478-86.

[Climo 2013] Climo MW, Yokoe DS, Warren DK, Perl TM, Bolon M, Herwaldt LA, Weinstein RA, Sepkowitz KA, Jernigan JA, Sanogo K, Wong ES. Effect of daily chlorhexidine bathing on hospital-acquired infection. N Engl J Med. 2013;368(6):533-42.

[Coulibaly 2013] Coulibaly YI, Dicko I, Keita M, Keita MM, Doumbia M, Daou A, Haidara FC, Sankare MH, Horton J, Whately-Smith C, Sow SO. A cluster randomized study of the safety of integrated treatment of trachoma and lymphatic filariasis in children and adults in Sikasso, Mali. PLoS Negl Trop Dis. 2013;7(5):e2221.

[Huang 2013] Huang SS, Septimus E, Kleinman K, Moody J, Hickok J, Avery TR, Lankiewicz J, Gombosev A, Terpstra L, Hartford F, Hayden MK, Jernigan JA, Weinstein RA, Fraser VJ, Haffenreffer K, Cui E, Kaganov RE, Lolans K, Perlin JB, Platt R. Targeted versus universal decolonization to prevent ICU infection. N Engl J Med. 2013;368(24):2255-65.

[Miller 2013] Miller S, Bergel EF, El Ayadi AM, Gibbons L, Butrick EA, Magwali T, Mkumba G, Kaseba C, Huong NT, Geissler JD, Merialdi M. Non-pneumatic anti-shock garment (NASG), a first-aid device to decrease maternal mortality from obstetric hemorrhage: a cluster randomized trial. PLoS One. 2013;8(10):e76477.

[Palmu 2013] Palmu AA, Jokinen J, Borys D, Nieminen H, Ruokokoski E, Siira L, Puumalainen T, Lommel P, Hezareh M, Moreira M, Schuerman L, Kilpi TM. Effectiveness of the ten-valent pneumococcal Haemophilus influenzae protein D conjugate vaccine (PHiD-CV10) against invasive pneumococcal disease: a cluster randomised trial. Lancet. 2013;381(9862):214-22.

[Pearce 2013] Pearce RJ, Ord R, Kaur H, Lupala C, Schellenberg J, Shirima K, Manzi F, Alonso P, Tanner M, Mshinda H, Roper C, Schellenberg D. A community-randomized evaluation of the effect of intermittent preventive treatment in infants on antimalarial drug resistance in southern Tanzania. J Infect Dis. 2013;207(5):848-59.

[Stanton 2013] Stanton CK, Newton S, Mullany LC, Cofie P, Tawiah Agyemang C, Adiibokah E, Amenga-Etego S, Darcy N, Khan S, Armbruster D, Gyapong J, Owusu-Agyei S. Effect on postpartum hemorrhage of prophylactic oxytocin (10 IU) by injection by community health officers in Ghana: a community-based, cluster-randomized trial. PLoS Med. 2013;10(10):e1001524.

[West 2013] West SK, Bailey R, Munoz B, Edwards T, Mkocha H, Gaydos C, Lietman T, Porco T, Mabey D, Quinn TC. A randomized trial of two coverage targets for mass treatment with azithromycin for trachoma. PLoS Negl Trop Dis. 2013;7(8):e2415.

[Churchyard 2014] Churchyard GJ, Fielding KL, Grant AD. A trial of mass isoniazid preventive therapy for tuberculosis control. N Engl J Med. 2014;370(17):1662-3.

[Cox 2014] Cox HS, Mbhele S, Mohess N, Whitelaw A, Muller O, Zemanay W, Little F, Azevedo V, Simpson J, Boehme CC, Nicol MP. Impact of Xpert MTB/RIF for TB diagnosis in a primary care clinic with high TB and HIV prevalence in South Africa: a pragmatic randomised trial. PLoS Med. 2014;11(11):e1001760.

[Juan-Giner 2014] Juan-Giner A, Domicent C, Langendorf C, Roper MH, Baoundoh P, Fermon F, Gakima P, Zipursky S, Tamadji M, Grais RF. A cluster randomized non-inferiority field trial on the immunogenicity and safety of tetanus toxoid vaccine kept in controlled temperature chain compared to cold chain. Vaccine. 2014;32(47):6220-6.

[Septimus 2014] Septimus EJ, Hayden MK, Kleinman K, Avery TR, Moody J, Weinstein RA, Hickok J, Lankiewicz J, Gombosev A, Haffenreffer K, Kaganov RE, Jernigan JA, Perlin JB, Platt R, Huang SS. Does chlorhexidine bathing in adult intensive care units reduce blood culture contamination? A pragmatic cluster-randomized trial. Infect Control Hosp Epidemiol. 2014;35 Suppl 3:S17-22.

[Jairath 2015] Jairath V, Kahan BC, Gray A, Dore CJ, Mora A, James MW, Stanley AJ, Everett SM, Bailey AA, Dallal H, Greenaway J, Le Jeune I, Darwent M, Church N, Reckless I, Hodge R, Dyer C, Meredith S, Llewelyn C, Palmer KR, Logan RF, Travis SP, Walsh TS, Murphy MF. Restrictive versus liberal blood transfusion for acute upper gastrointestinal bleeding (TRIGGER): a pragmatic, open-label, cluster randomised feasibility trial. Lancet. 2015;386(9989):137-44.

[Juthani-Mehta 2015] Juthani-Mehta M, Van Ness PH, McGloin J, Argraves S, Chen S, Charpentier P, Miller L, Williams K, Wall D, Baker D, Tinetti M, Peduzzi P, Quagliarello VJ. A cluster-randomized controlled trial of a multicomponent intervention protocol for pneumonia prevention among nursing home elders. Clin Infect Dis. 2015;60(6):849-57.

[Kalra 2015] Kalra L, Irshad S, Hodsoll J, Simpson M, Gulliford M, Smithard D, Patel A, Rebollo-Mesa I. Prophylactic antibiotics after acute stroke for reducing pneumonia in patients with dysphagia (STROKE-INF): a prospective, cluster-randomised, open-label, masked endpoint, controlled clinical trial. Lancet. 2015;386(10006):1835-44.

[Ononge 2015] Ononge S, Campbell OM, Kaharuza F, Lewis JJ, Fielding K, Mirembe F. Effectiveness and safety of misoprostol distributed to antenatal women to prevent postpartum haemorrhage after child-births: a stepped-wedge cluster-randomized trial. BMC Pregnancy Childbirth. 2015;15:315.

[Postma 2015] Postma DF, van Werkhoven CH, van Elden LJR, Thijsen SFT, Hoepelman AIM, Kluytmans JAJW, Boersma WG, Compaijen CJ, van der Wall E, Prins JM, Oosterheert JJ, Bonten MJM. Antibiotic Treatment Strategies for Community-Acquired Pneumonia in Adults. New England Journal of Medicine. 2015;372(14):1312-23.

[Karkouti 2016] Karkouti K, Callum J, Wijeysundera DN, Rao V, Crowther M, Grocott HP, Pinto R, Scales DC. Point-of-Care Hemostatic Testing in Cardiac Surgery: A Stepped-Wedge Clustered Randomized Controlled Trial. Circulation. 2016;134(16):1152-62.

[Long 2016] Long JE, Drayson MT, Taylor AE, Toellner KM, Lord JM, Phillips AC. Morning vaccination enhances antibody response over afternoon vaccination: A cluster-randomised trial. Vaccine. 2016;34(24):2679-85.

**Supplementary Material 1: Data abstraction form**

**Baseline characteristics**

1. **Publication year** (Categorical)

- 2007-2010
- 2011-2013
- 2014-2016

1. **What is the country in which the study has taken place?** (Categorical)

- Canada
- USA
- Canada and USA
- United Kingdom
- Elsewhere in the European Union
- Australia
- Low Middle Income Countries (LMICs)

1. **What type of setting is this study from?** (Binary)

- Healthcare
- Non-healthcare

1. **What is the cluster defined as?** (Categorical)

- Residential areas
- Primary care practices
- Individual health professionals
- Hospitals
- Nursing homes
- Medical clinics
- Intensive Care Units
- Other
- If other please specify

1. **What is the total number of realised clusters used in this study?** (Numerical)

Notes- Realised clusters are clusters that were recruited and baseline measures were taken, i.e. that did not drop out prior to baseline measures being taken. If unclear please leave blank.

1. **What is the total number of realised participants for this study?** (Numerical)

Notes- Realised participants are participants that were recruited and baseline measures were taken, i.e. that did not drop out prior to baseline measures taken. If unclear please leave blank.

1. **Reported presence of a Data Safety Monitoring Committee (DSMC)** (Binary)
2. **Self-identified as pragmatic trial** (Binary)
3. **Research ethics review reported** (Binary)

**Section 0 – Demographics**

This section seeks to gather information on general cluster study information**.**

1. What rationale was reported for choosing a cluster trial design?

- Avoid contamination
- Practical reasons
- Cluster level intervention
- Unclear
- Other
- If other, Please give further details (Free text- compulsory to answer)
- If unclear, please give further details (free text – optional)

1. How many eligibility criteria were there for the inclusion of **clusters**?

- Less than 3
- Equal to or greater than 3
- Unclear
- If unclear, please give further details (free text – optional)

1. How many eligibility criteria were there for the inclusion of **participants**?

- Less than 3
- Equal to or greater than 3
- Unclear
- If unclear, please give further details (free text – optional)

**Section 1 – Bias arising from the randomisation process**

This section contains questions to gather information about the randomisation process, including aspects on concealment of the allocation to intervention and control groups and recruitment.

1. What is the trial design?

- Parallel arm
- Factorial
- Cross-over
- Stepped wedge

1. How was the randomisation of **clusters** to allocated treatment(s) conducted? (Tick all that apply)

**Note-** If the only information about randomisation methods is to state that the cluster is randomised, then this question should generally be answered as 'Other' and detail that this is the case.

- Random number generator
- Random number table
- Simple randomisation
- Minimisation (with and without a random element)
- Block randomisation
- Stratified
- Restricted/constrained
- Pair-matched
- Unclear
- Other
- If other, Please give further details (Free text- compulsory to answer)
- If unclear, please give further details (free text – optional)

1. Is there any mention of methods you might think are non-random?

- Yes
- No
- Unclear
- If unclear, please give further details (free text – optional)

1. If yes or unclear in question 3, what non-random allocation method was used?

- Alternation
- Method based on dates (e.g. of birth or hospital admission)
- Patient record numbers
- Allocation decision made by clinicians or participants
- Based on the availability of the intervention
- Any other systematic or haphazard method
- Unclear
- Other
- If other, Please give further details (Free text- compulsory to answer)
- If unclear, please give further details (free text – optional)

1. For stepped wedge trial, was there any indication that some clusters were preferentially allocated to a specific sequence in a non-random way?

**Notes:** Were any clusters not randomly allocated to a sequence, perhaps for example because it was felt that there was a need for some clusters to receive the intervention first

- Yes
- No
- Unclear
- If unclear, please give further details (free text – optional)

1. Who conducted the randomisation?

- Independent researcher from a trials unit
- Independent researcher (other)
- Someone within the trial team
- Unclear
- Other
- If other, Please give further details (Free text- compulsory to answer)
- If unclear, please give further details (free text – optional)

1. How was the randomisation allocation of **clusters** concealed?

- Tamper-proof sealed or opaque envelop
- Telephone
- Internet-based randomisation service
- Unclear
- Other
- If other, Please give further details (Free text- compulsory to answer)
- If unclear, please give further details (free text – optional)

1. Were **all** clusters recruited before randomisation?

- Yes
- No
- Unclear
- If unclear, please give further details (free text – optional)

1. If No or Unclear answered in question 8, when were clusters recruited?

- After randomisation
- Before and after randomisation
- Unclear
- If unclear, please give further details (free text – optional)

1. Were there baseline imbalances across **cluster level characteristics** to suggest a problem with the randomisation process?

**Note** –

- This question is specifically looking at **cluster level characteristics** not participant level characteristics
- Answer ‘No’ if no imbalances are apparent or if any observed imbalances are compatible with chance
- Answer ‘Yes’ only if there is clear evidence of imbalances that appear to be due to problems with randomisation
- If there is no information about cluster characteristics record ‘No information’
- Yes
- No
- No information on cluster level characteristics
- Unclear
- If yes, please give further details on the imbalance

**For example**, when assessing cluster level characteristics where the clusters are hospitals, there could be more clusters in the intervention condition that have an Accident and Emergency department compared to the clusters in the control condition. Thus an imbalance at the cluster level characteristics, specifically in this case if the cluster (hospital) had an Accident and Emergency department or not. (Free text - compulsory)

- If unclear, please give further details (free text - optional)

1. If question 1 is answered ‘Stepped wedge’; when were timings of transitions (i.e. the time when the cluster moves from control to treatment) revealed to clusters?

- Start of the study
- Pre-specified time before cluster is due to receive the intervention
- Unclear
- Other
- If other, Please give further details (Free text- compulsory to answer)
- If unclear, please give further details (free text – optional)

**Section 2 - Bias arising from the timing of identification and recruitment of individual participants in relation to timing of randomisation**

This section contains questions on how participants were identified and recruited into the study.

**NOTES:** Definition of participants:

For the purpose of this data extraction, **participants** are defined as those individuals on whom it has been decided to collect data for the outcome of interest (Eldridge, S., et al. (2016) *Revised Cochrane risk of bias tool for randomized trials (RoB2.0) Additional considerations for cluster randomized trials.* Accessed:16/5/2019. <https://sites.google.com/site/riskofbiastool/welcome/rob-2-0-tool/archive-rob-2-0-cluster-randomized-trials-2016>).

**For example:**

In a study where the primary outcome is assessing mortality of patients, the **participant** here is the patient. Whereas, in a study where the primary outcome relates to a survey on health care professionals, the **participant** here is the health care professional not the patient interacting with the health care professionals.

1. Were participants **recruited** to the study?

- Yes
- No
- Unclear
- If unclear, please give further details (free text – optional)

**If no or unclear has been answered in *question one* please complete the following questions:**

1. When were the **study** participants **identified** for inclusion to the study?

Note: Participants that do not need to be informed that they are in a study, i.e. retrospective assessment of GP records, are still study participants.

- Before randomisation of clusters
- After randomisation of clusters
- Before and after randomisation of clusters
- Unclear
- If unclear, please give further details (free text – optional)

1. If in question 2 ‘After randomisation of clusters’, ‘Before and after randomisation’ or ‘Unclear’ was selected, were those **identifying** study participants aware of the cluster allocation?

Note: Participants that do not need to be informed that they are in a study, i.e. retrospective assessment of GP records, are still study participants.

- Yes
- No
- Unclear
- If unclear, please give further details (free text – optional)

1. If no or unclear to question 3, how were the cluster allocations concealed? Please provide a quote.

Free text – compulsory to fill in

1. If in question 2 ‘After randomisation of clusters’, ‘Before and after randomisation’ or ‘unclear’ was selected, were **actual** **participants** themselves aware of the cluster allocation?

- Yes
- No
- Unclear
- If unclear, please give further details (free text – optional)

**If yes has been answered in *question one* please complete the following questions:**

1. When were the **potential** participants **identified** for inclusion to the study?

- Before randomisation of clusters
- After randomisation of clusters
- Before and after randomisation of clusters
- Unclear
- If unclear, please give further details (free text – optional)

1. If in question 6 ‘After randomisation of clusters’, ‘Before and after randomisation’ or ‘Unclear’ was selected, were those **identifying** these **potential** participants aware of the cluster allocation?

Note: Participants that meet the eligibility criteria of the study and are required to be invited or consented to take part in the study, i.e. those that have yet to be confirmed to participate, are defined as **potential** participants.

- Yes
- No
- Unclear
- If unclear, please give further details (free text – optional)

1. If no or unclear in question 7, how were the cluster allocations concealed to those **identifying** these **potential** participants? Please provide a quote.

Free text – compulsory to fill in

1. When were the **potential** participants **recruited** for inclusion to the study?

- Before randomisation of clusters
- After randomisation of clusters
- Before and after randomisation
- Unclear
- If unclear, please give further details (free text – optional)

1. If in question 9 ‘After randomisation of clusters’, ‘Before and after randomisation’ or ‘Unclear’ was selected, were those **recruiting** **participants** aware of the cluster allocation?

- Yes
- No
- Unclear
- If unclear, please give further details (free text – optional)

1. In question 10 if no or unclear, how were the cluster allocations concealed to those **recruiting participants**? Please provide a quote.

**For example,** study personnel recruiting participants had access to files detailing cluster allocation.

Free text – compulsory to fill in

1. If in question 6 or 9 ‘After randomisation of clusters’, ‘Before and after randomisation’ or ‘unclear’ was selected, were **actual** **participants** themselves aware of the cluster allocation?

- Yes
- No
- Unclear
- If unclear, please give further details (free text – optional)

1. In question 12 if no or unclear, how were the cluster allocations concealed from actual participants? Please provide a quote.

Free text – compulsory to fill in

1. Were there baseline imbalances that suggest differential identification or recruitment of **individual participants** between arms?

Note –

- Answer ‘No’ if no imbalances are apparent or if any observed imbalances are compatible with chance
- Answer ‘Yes’ only if there is clear evidence of imbalances that appear to be due to problems with randomisation at cluster level
- If there is no information about participant level characteristics record ‘No information’
- Yes
- No
- No information on individual participants
- Unclear
- If unclear, please give further details (free text – optional)
- If no or no information, please give further details (Free text - compulsory)
- If yes, please give further details (Free text - compulsory)

**Section 3 – Bias due to deviations from intended intervention**

This section contains questions relating to the participants awareness of study participation and study hypotheses.

NOTES: Definition of participants:

For the purpose of this data extraction **participants** are defined as those individuals on whom it has been decided to collect data for the outcome of interest (Eldridge, S., et al. (2016) *Revised Cochrane risk of bias tool for randomized trials (RoB2.0) Additional considerations for cluster randomized trials.* Accessed:16/5/2019. <https://sites.google.com/site/riskofbiastool/welcome/rob-2-0-tool/archive-rob-2-0-cluster-randomized-trials-2016>).

**For example:**

In a study where the primary outcome is assessing mortality of patients, the **participant** here is the patient. Whereas, in a study where the primary outcome relates to a survey on health care professionals the **participant** here is the health care professional not the patient interacting with the health care professionals.

1. Were participants aware that they were in a trial?

- Yes
- No
- Unclear
- If unclear, please give further details (free text – optional)

1. If yes or unclear to question 1, did any participants know of the intervention(s) being compared in the study?

- Yes
- No
- Unclear
- If unclear, please give further details (free text – optional)

1. If yes or unclear has been answered for question 1, were any participants aware of their assigned intervention during the trial?

- Yes
- No
- Unclear
- If unclear, please give further details (free text – optional)

1. Were any people who had interactions with the participants (i.e. nurses, doctors, family, trial personnel) aware of participants' assigned intervention during the trial?

- Yes
- No
- Unclear
- If unclear, please give further details (free text – optional)

1. Was there evidence to suggest that there were strong deviations from the intended control condition? **For example,** participants in the control condition being provided with the intervention condition.

- Yes
- No
- Unclear
- If unclear, please give further details (free text – optional)

1. Was there evidence to suggest that the intervention was **not** delivered as expected?

**For example,** if the intervention was to introduce exercise classes twice a week for a month to the intervention participants, was there a substantial number of intended exercise classes not run over the course of the month.

- Yes
- No
- Unclear
- If unclear, please give further details (free text – optional)

1. If ‘yes’ is answered in question 5 or question 6, Were these deviations from intended intervention unbalanced between arms *and* likely to have affected the outcome?

- Yes
- No
- Unclear
- If unclear, please give further details (free text – optional)

1. For the primary outcome, were any **clusters** analysed in an assigned intervention different from the one to which they were assigned to?

- Yes
- No
- Unclear
- If unclear, please give further details (free text – optional)

1. If yes or unclear answered in question 8, were more than about 5% of **clusters** analysed in an assigned intervention different from the one to which they were assigned to?

- Yes
- No
- Unclear
- If unclear, please give further details (free text – optional)

1. For the primary outcome, were any **participants** analysed in an assigned intervention different from the one to which they were assigned to?

- Yes
- No
- Unclear
- If unclear, please give further details (free text – optional)

1. If yes, or unclear answered in question 10, were more than about 5% of **participants** analysed in an assigned intervention different from the one to which they were assigned to?

- Yes
- No
- Unclear
- If unclear, please give further details (free text – optional)

1. If stepped wedge or cross over design, Did clusters transition to the intervention condition when they were planned to (according to the randomisation schedule)?

- Yes
- No
- Unclear

1. If stepped wedge or cross over design, was a wash out period or transition period used?

Notes:

A transition period are defined as a period of time between the control and intervention delivery whereby to allow for the intervention to be fully implemented.

A washout period is defined as a period of time between the intervention and control so the intervention can be withdrawn.

- Yes
- No
- Unclear
- If unclear, please give further details (free text – optional)

1. If stepped wedge or crossover design, was a justification provided for the length of this washout or transition period (including zero length)?

- Yes
- No
- Unclear
- If unclear, please give further details (free text – optional)

1. If yes, please provide a quote from the paper.
2. If stepped wedge or cross over design, was there a delayed assessment of primary outcome and a long exposure to intervention?

- **For example,** a trial evaluating the impact of a change in the way a service is delivered in a nursing home, patients might have a long exposure to a treatment (i.e. they become exposed to the other treatment condition when the cluster switches treatment condition) AND would have a delayed assessment of the outcome (i.e. outcome assessed now in the other treatment condition) if outcome was length of stay in a nursing home
- Yes
- No
- Unclear
- If unclear, please give further details (free text – optional)

1. If yes in question 16, If any participants were exposed to both the intervention and control conditions was a time dependent treatment covariate included in the analysis

**Notes:** For example, a patient still in the study from control to intervention condition, and treatment indicator to account for switching to intervention condition.

- Yes
- No
- Unclear
- If unclear, Please give further details on the answer you selected on if a time dependent treatment covariate was included in the analysis. (free text – optional)
- If Yes, Please give further details on the answer you selected on if a time dependent treatment covariate was included in the analysis. (free text – optional)
- If No, Please give further details on the answer you selected on if a time dependent treatment covariate was included in the analysis. (free text – optional)

**Section 4 – Bias due to missing outcome data**

This section contains questions relating to the handling of missing data at both cluster and participant level and the subsequent steps taken in the analysis.

In this section please refer to the primary outcome unless otherwise specified please answer with respect to the primary analysis (the main analysis set out in the methods).

1. For the primary outcome, did more than approximately 90% of randomised **clusters** contribute to the **primary analysis**?

- Yes
- No
- Unclear
- If unclear, please give further details (free text – optional)

1. For the primary outcome, what is the percentage of **clusters** that contributed to the **primary analysis**? (Optional)

- % (range from 0 to 100 inclusive)

1. For the **primary outcome**, were outcome data available for more than approximately 90% of **participants**?

- Yes
- No
- Unclear
- If unclear, please give further details (free text – optional)

1. For the primary outcome, what is the percentage of **participants** that contributed to the **primary analysis**? (Optional)

- % (range from 0 to 100 inclusive)

1. If unclear or no in question 1, are the **proportions** of missing **primary** outcome data similar across control and intervention conditions for **clusters**?

- Yes
- No
- Unclear
- If unclear, please give further details (free text – optional)

1. If unclear or no in question 1, are the **reasons** for missing **primary** outcome data similar across control and intervention conditions for **clusters**?

- Yes
- No
- Unclear
- If unclear, please give further details (free text – optional)

1. If unclear or no in question 3, are the **proportions** of missing **primary** outcome data similar across control and intervention conditions for **participants**?

- Yes
- No
- Unclear
- If unclear, please give further details (free text – optional)

1. If unclear or no in question 3, are the **reasons** for missing **primary** outcome data similar across control and intervention conditions for **participants**?

- Yes
- No
- Unclear
- If unclear, please give further details (free text – optional)

1. If **no or unclear** in questions 1 or 3, how were missing data handled in the **primary** analysis?

- Multiple imputation
- Complete case
- Unclear
- Other
- If other, Please give further details (Free text- compulsory to answer)
- If unclear, please give further details (free text – optional)

1. For the primary outcome, were other analysis reported, if so what was reported? (tick all that apply)

- No other analysis reported
- Multiple imputation
- Complete case
- Sensitivity analysis
- Unclear
- Other
- If other, Please give further details (Free text- compulsory to answer)
- If unclear, please give further details (free text – optional)

1. If ‘no other analysis reported’ in question 10 do not answer this question, were the results between the primary analysis and these sensitivity analyses for the primary outcome similar?

- The results were similar
- The results were not similar
- Unclear
- If unclear, please give further details (free text – optional)

**Section 5 – Risk of bias in measurement of the outcome**

This section contains questions that are in relation to how the primary outcome is measured.

1. Was the outcome subjective or objective?

For example, a subjective outcome could be a patient reported outcome such as pain score. An objective outcome could be blood pressure or death.

- Objective
- Subjective
- Unclear
- If unclear, please give further details (free text – not compulsory)

1. Was the participant the outcome assessor?

- Yes
- No
- Unclear
- If unclear, please give further details (free text – optional)

1. Were the primary outcome assessors aware that a trial was taking place?

- Yes
- No
- Unclear
- If unclear, please give further details (free text – optional)

1. Were the primary outcome assessors aware of the intervention received by the **cluster**?

- Yes
- No
- Unclear
- If unclear, please give further details (free text – optional)

1. If no or unclear to question 4, how were the **cluster** allocations concealed from the primary outcome assessors? Please provide a quote.

Free text – compulsory to fill in

1. Were the primary outcome assessors aware of the intervention received by the **participant**?

- Yes
- No
- Unclear
- If unclear, please give further details (free text – optional)

1. If no or unclear to question 6, how were the cluster allocations concealed from the primary outcome assessors? Please provide a quote.

Free text – compulsory to fill in

**Section 6 – Bias in selection of the reported result**

This section contains questions about whether the results were reported as intended as well as how they were actually reported.

**Part A: For the following questions, assess the planned analysis within the methods section *or* protocol paper (if provided) specifically looking at the primary outcome.**

1. Has the primary outcome been pre-specified?

- Yes
- No
- Unclear
- If unclear, please give further details (free-text – optional)

1. For the primary outcome only, was an assessment time specified?

Note: The assessment time is defined as the time at which the primary outcome is measured at for the primary analysis. For example, the outcome measure was recorded at 3, 6 and 12 months. The primary outcome measure was pre-specified to be at 12 months.

- Yes
- No
- Unclear
- If unclear, please give further details (free text – optional)

1. Was it detailed whether the **primary outcome** results would be reported as adjusted or unadjusted (except for clustering)?

Notes: For example, adjustment for other characteristics that could influence the outcome.

- Yes
- No
- Unclear
- If unclear, please give further details (free text – optional)

1. For the primary outcome, was it detailed that the analysis would account for clustering?

- Yes the analysis planned accounted for clustering
- No the analysis planned did not account for clustering
- Unclear
- If unclear, please give further details (free text – optional)

1. For the primary outcome, were there pre-specified methods given on how to handle missing data for the primary analysis?

- Yes
- No
- Unclear
- If unclear, please give further details (free text – optional)

1. Were there details on what scale the primary outcome results would be given on?

Notes: For example, HbA1c can be measured and recorded as a percentage or as mmol/mol.

- Yes
- No
- Unclear
- If unclear, please give further details (free text – optional)

1. If the primary outcome measure was initially a **continuous variable** but for the purpose of this study it was **dichotomised or categorised**, was it clear what cut point was going to be used?

- The primary outcome measure was not continuous
- Yes
- No
- Unclear
- If unclear, please give further details (free text – optional)

**Part B: For the next question please assess the realised analysis (i.e. results sections) specifically for the primary outcome.**

1. For the primary outcome, how were the results reported? (check all that apply)

- Unadjusted (except for clustering)
- Unadjusted (without clustering)
- Adjusted (for other covariates)
- Partially adjusted (for a subset of the covariates specified in methods)
- Transformed
- Complete case
- After multiple imputation
- At different cut points
- On different scales
- At multiple time points
- Unclear
- Other
- If other, Please give further details (Free text- compulsory to answer)
- If unclear, please give further details (free text – optional)

**Section 7 – Risk of bias arising from the statistical analysis**

This section contains questions about the specific statistical analysis that has been used given the study design.

1. For the primary outcome, what method was used to adjust for clustering in the **primary** analysis?

- None
- Mixed model
- Generalised estimating equation (GEE) (marginal model)
- Robust variance estimation
- Cluster level analysis
- Unclear
- Other
- If other, Please give further details (Free text- compulsory to answer)
- If unclear, please give further details (free text – optional)

1. For the primary outcome, was a small sample correction used in the primary analysis?

Note: By a small sample correction we mean a small adjustment made to the standard errors for treatment effects, recommended when there are a small number of clusters

- Yes
- No
- Unclear
- If unclear, please give further details (free text – optional)

1. If yes is answered in question 2 and ‘Mixed model’ was selected in question 1, what degrees of freedom correction was used?

- Between-within
- Kenwood-roger
- Saitterthwaite
- Unclear
- If unclear, please give further details (free text – optional)

1. If yes is answered in question 2 and ‘Generalised estimating equations (GEE)’ was selected in question 1, what standard error small sample correction was used?

- Fay and Graubard (R package saws may be mentioned)
- Kauermann and Carroll
- Mackinnon and white (clusters – parameters)
- Mancl and DeRouen
- Morel, Bokossa, and Neerchal
- None
- Unclear
- Other
- If other, Please give further details (Free text- compulsory to answer)
- If unclear, please give further details (free text – optional)

1. If yes is answered in question 2 and ‘Generalised estimating equations (GEE)’ was selected in question 1, what degrees of freedom correction was used?

- Fay and Graubard
- Clusters – parameters
- None
- Unclear
- Other
- If other, please give further details (free text – compulsory)
- If unclear, please give further details. (free text – optional)

1. For a stepped wedge or crossover trial design: For the **primary outcome** was time adjusted for in the primary analysis?

- Yes
- No
- Unclear
- If unclear, please give further details (free text – optional)

1. If yes (or unclear) is answered for question 6, what method had been used to account for time effect?

- Assuming a linear time trend
- Fixed effect for each time period
- Linear time trend and random slope for each cluster
- Other more complex forms (e.g. cubic trends etc)
- Unclear
- Other
- If other, Please give further details (Free text- compulsory to answer)
- If unclear, please give further details (free text – optional)

Supplementary Material 2: Mapping of signalling questions and data extraction questions

| **RoB2.0 signalling question** | **Data extraction question** | **Data extraction answer** | **Comment** | **Standalone what this indicates in signalling question** | **Signalling question answer (given combinations of possible answers)** |
| --- | --- | --- | --- | --- | --- |
| 1a.1 Was the allocation sequence random? | 1. How was the randomisation of clusters to allocated treatment(s) conducted? (Tick all that apply) | - Random number generator - Random number table - Simple randomisation - Minimisation (with or without a random element) - Block randomisation - Stratified - Restricted/constrained - Pair-matched - Other - If other, Please give further details (Free text- compulsory to answer) | Any method (or combination of methods) selected | Indicates Yes in signalling question **(a)** | **Yes**  If: **(a)** or **(A)**  or  If **(b)** and **(d)**  **No information**  If:  **(b)** and **(e)** and **(g)**  **No**  If: **(b)** and **(c)**  or  If: **(b)** and **(e)** and **(f)**  **Check back in the paper:**  If: [**(a)** or **(A)**] and **(c)**  or  If: [**(a)** or **(A)**] and **(e)** and **(f)** |
|  |  | - Simple randomisation - Other   If other, Please give further details (Free text- compulsory to answer) | If simple randomisation alone is selected or if in other states that ‘randomly’ or ‘randomisation’ is just used and no method has been given | Indicates Yes in signalling question **(A)** |  |
|  |  | - Unclear - If unclear, please give further details (free text – optional) | If no information is indicated | Indicates No information in signalling question **(b)** |  |
|  | 1. Is there any mention of methods you might think are non-random? | - Yes |  | Indicates No in signalling question **(c)** |  |
|  |  | - No |  | Indicates Yes in signalling question **(d)** |  |
|  |  | - Unclear - If unclear, please give further details (free text – optional) | If no information is indicated | Indicates No information in signalling question **(e)** |  |
|  | 1. If yes or unclear in question 3, what non-random allocation method was used? | - Alternation - Method based on dates (e.g. of birth or hospital admission) - Patient record numbers - Allocation decision made by clinicians or participants - Based on the availability of the intervention - Any other systematic or haphazard method - Other - If other, Please give further details (Free text- compulsory to answer) | Any answer | Indicates No in signalling question **(f)** |  |
|  |  | - Unclear - If unclear, please give further details (free text – optional) | If no information is indicated | Indicates No information in signalling question **(g)** |  |
| 1a.2 Is it likely that the allocation sequence was subverted? | 1. Who conducted the randomisation? | - Independent researcher from a trials unit - Independent researcher (other) | Either of these selected | Indicates No in signalling question **(h)** | **Yes**  If: not {**(h)** and [**(k)** or **(m)**]}  **No information**  If:  **(j)** & **(l)**  **No**  If**: (h)** or [**(k)** or **(m)**]  Questions 8 and 9 are for more details and not used directly in the mapping. |
|  |  | - Someone within the trial team |  | Indicates Yes in signalling question **(i)** |  |
|  |  | - Unclear - If unclear, please give further details (free text – optional) | If no information is indicated | Indicates No information in signalling question **(j)** |  |
|  |  | - Other - If other, Please give further details (Free text- compulsory to answer) | Case by case basis | Dependent on answer |  |
|  | 1. How was the randomisation allocation of clusters concealed? | - Tamper-proof sealed or opaque envelop - Telephone - Internet-based randomisation service | Either of these selected | Indicates No in signalling question **(k)** |  |
|  |  | - Unclear - If unclear, please give further details (free text – optional) | If no information is indicated | Indicates No information in signalling question **(l)** |  |
|  |  | - Other - If other, Please give further details (Free text- compulsory to answer) | If indicated that conceal but method was not detailed | Indicated No in the signalling question **(m)** |  |
|  |  |  | If indicated that the allocation was not concealed | Indicated Yes in the signalling question **(n)** |  |
|  | 1. Were all clusters recruited before randomisation? | - Yes |  | Indicates No in signalling question **(o)** |  |
|  |  | - No |  | Indicated Yes in the signalling question **(p)** |  |
|  |  | - Unclear - If unclear, please give further details (free text – optional) | If no information is indicated | Indicated No information in the signalling question **(q)** |  |
|  | 1. If No or Unclear answered in question 8, when were clusters recruited? | - After randomisation - Before and after randomisation |  | Indicated Yes in the signalling question **(r)** |  |
|  |  | - Unclear   If unclear, please give further details (free text – optional) | If no information is indicated | Indicated No information in the signalling question **(s)** |  |
| 1a.3 Were there baseline imbalances that suggest a problem with the randomization process? | 1. Were there baseline imbalances across cluster level characteristics to suggest a problem with the randomisation process? | - Yes - If yes, please give further details on the imbalance (Free text - compulsory) |  | Indicated Yes information in the signalling question **(t)** | **Yes:**  If **(t)**  **No information:**  If **(v)** or **(w)**  **No:**  If **(u)** |
|  |  | - No |  | Indicated No in the signalling question **(u)** |  |
|  |  | - No information on cluster level characteristics |  | Indicated No information in the signalling question **(v)** |  |
|  |  | - Unclear - If unclear, please give further details (free text - optional) | If no information or not enough information is indicated | Indicated No information in the signalling question **(w)** |  |

|  | | | | | |
| --- | --- | --- | --- | --- | --- |
| **RoB2.0 Signalling questions** | **Data extraction question** | **Data extraction answer** | **Comment** | **Standalone what this indicates in signalling question** | **Signalling question**  **answer (given combinations of possible answers)** |
| 1b.1 Were all the individual participants identified before randomization of clusters (and if the trial specifically recruited patients were they all recruited before randomization of clusters)? | **If no or unclear has been answered in *question one* please complete the following questions:**   1. When were the **study** participants **identified** for inclusion to the study? | - Before randomisation of clusters |  | Indicates Yes in this signalling question **(a)** | **If no or unclear in Question 1** (i.e. participants were NOT recruited for the study)  **Yes**  If: **(a)** only  **No**  If: **(b)** only  **No information**  If: **(c)** only  **If Yes in Question 1** (i.e. participants were recruited for the study)  **Yes**  If: **(d)** & **(g)**  **No**  If: **(e)** or **(h)**  **No information**  If: **(f)** & **(i)** |
|  |  | - After randomisation of clusters - Before and after randomisation of clusters | Either | Indicates No in this signalling question **(b)** |  |
|  |  | - Unclear - If unclear, please give further details (free text – optional) | If indicates there is no information | Indicates no information  **(c)** |  |
|  | **If yes has been answered in *question one* please complete the following questions:**   1. When were the **potential** participants **identified** for inclusion to the study? | - Before randomisation of clusters |  | This indicates Yes in the signalling question but needs to be entered for Question 9 also **(d)** |  |
|  |  | - After randomisation of clusters - Before and after randomisation of clusters | Either | Indicates No in this signalling question  **(e)** |  |
|  |  | - Unclear - If unclear, please give further details (free text – optional) | If indicates there is no information | Indicates no information  And q9 would need to be unclear too to give NI for this signalling Question  **(f)** |  |
|  | **If yes has been answered in *question one* please complete the following questions:**   1. When were the **potential** participants **recruited** for inclusion to the study? | - Before randomisation of clusters |  | This indicated Yes in the signalling question (as Q6 should also have been answered like this) **(g)** |  |
|  |  | - After randomisation of clusters - Before and after randomisation | Either | Indicates No in this signalling question  Irrespective of what is answered in Q6  **(h)** |  |
|  |  | - Unclear ( If unclear, please give further details (free text – optional)) | If indicates there is no information | Indicates no information  **(i)** |  |
| 1b.2 If N/PN/NI to 1b.1: Is it likely that selection of individual participants was  affected by knowledge of the intervention? | **If no or unclear has been answered in *question one* please complete the following questions (i.e., no participant recruitment, or no information on participant recruitment):**   1. If in question 2 ‘After randomisation of clusters’, ‘Before and after randomisation’ or ‘Unclear’ was selected, were those **identifying** study participants aware of the cluster allocation? | - Yes |  | Yes in the signalling question **(j)** | **If no or unclear in Q1** (i.e. participants were NOT recruited for the study)  **No**  If answer to question 1 is no (i.e. no recruitment or participant consent) answer to 1b.2 is no.  **No information**  If answer to question 1 is no information (i.e. no information on recruitment or participant consent) answer to 1b.2 is no information.  **For when question 1b.1 is answer No or unclear:**  **If Yes in Q1** (i.e. participants were recruited for the study)  **Yes**  If: any combination except **(q)** and **(t)** and **(w);** or **(r)** and **(u)** and **(x).**  **No**  If: **(q)** and **(t)** and **(w)** together only  **No information**  If: **(r)** and **(u)** and **(x)** together only |
|  |  | - No |  | No in the signalling question if Q5 is also No **(k)** |  |
|  |  | - Unclear - If unclear, please give further details (free text – optional) |  | Indicates no information **(l)** |  |
|  | 1. If no or unclear to question 3, how were the cluster allocations concealed? Please provide a quote. | Free text – compulsory to fill in |  |  |  |
|  | 1. If in question 2 ‘After randomisation of clusters’, ‘Before and after randomisation’ or ‘unclear’ was selected, were **actual** **participants** themselves aware of the cluster allocation? | - Yes |  | Yes in the signalling question **(m)** |  |
|  |  | - No |  | No in the signalling question if Q3 is also No **(n)** |  |
|  |  | - Unclear - If unclear, please give further details (free text – optional) |  | Indicates no information **(o)** |  |
|  | **If yes has been answered in *question one* please complete the following questions:**   1. If in question 6 ‘After randomisation of clusters’, ‘Before and after randomisation’ or ‘Unclear’ was selected, were those **identifying** these **potential** participants aware of the cluster allocation? | - Yes |  | Yes in the signalling question **(p)** |  |
|  |  | - No |  | No in the signalling question if Q10 and Q12 are also No **(q)** |  |
|  |  | - Unclear - If unclear, please give further details (free text – optional) |  | Indicates no information **(r)** |  |
|  | 1. If no or unclear in question 7, how were the cluster allocations concealed? Please provide a quote. | Free text – compulsory to fill in |  |  |  |
|  | 1. If in question 9 ‘After randomisation of clusters’, ‘Before and after randomisation’ or ‘Unclear’ was selected, were those **recruiting** **participants** aware of the cluster allocation? | - Yes |  | Yes in the signalling question **(s)** |  |
|  |  | - No |  | No in the signalling question if Q7 and Q12 are also No **(t)** |  |
|  |  | - Unclear   If unclear, please give further details (free text – optional) |  | Indicates no information **(u)** |  |
|  | 1. In question 10 if no or unclear, how were the cluster allocations concealed? Please provide a quote. | Free text – compulsory to fill in |  |  |  |
|  | 1. If in question 6 or 9 ‘After randomisation of clusters’, ‘Before and after randomisation’ or ‘unclear’ was selected, were **actual** **participants** themselves aware of the cluster allocation? | - Yes |  | Yes in the signalling question **(v)** |  |
|  |  | - No |  | No in the signalling question if Q10 and Q7 are also No **(w)** |  |
|  |  | - Unclear - If unclear, please give further details (free text – optional) |  | Indicates no information **(x)** |  |
|  | 1. In question 12 if no or unclear, how were the cluster allocations concealed? Please provide a quote. | Free text – compulsory to fill in |  |  |  |
| 1b.3 Were there baseline imbalances that suggest differential identification or recruitment of individual participants between arms? | 1. Were there baseline imbalances that suggest differential identification or recruitment of individual participants between arms? | - Yes - If yes, please give further details (Free text - compulsory) |  | Yes in the signalling question **(y)** | **Yes**  If: **(y)**  **No**  If: **(Y)**  **No information**  If: **(z)** or **(Z)** |
|  |  | - No - If no or no information, please give further details (Free text - compulsory) |  | No in the signalling question **(Y)** |  |
|  |  | - No information on individual participants - If no or no information, please give further details (Free text - compulsory) |  | Indicates no information **(z)** |  |
|  |  | - Unclear - If unclear, please give further details (free text – optional) |  | Indicates no information **(Z)** |  |

| **RoB2.0 Signalling questions** | **Data extraction form questions** | **Data extraction answer** | **Comment** | **Standalone what this indicates in signalling question** | **Signalling question answer (given combinations of possible answers)** |
| --- | --- | --- | --- | --- | --- |
| 2.1a Were participants aware that they were in a trial? | 1. Were participants aware that they were in a trial? | - Yes |  | Yes in the signalling question **(a)** | One to one with the signalling question  Yes: **(a)**  No: **(b)**  No information: **(c)** |
|  |  | - No |  | No in the signalling question  **(b)** |  |
|  |  | - Unclear - If unclear, please give further details (free text – optional) | If no information is implied | No information in the signalling question **(c)** |  |
| 2.1b If Y/PY/NI to 2.1a: Were participants aware of their assigned intervention during the trial? | 1. If yes or unclear to question 1, did any participants know of the intervention(s) being compared in the study? | - Yes |  | Yes in the signalling question **(d)** | Just using Question 3; one to one with the signalling question  Yes: **(g)**  No: **(h)**  No information: **(i)**  Question 2 is for extra information. |
|  |  | - No |  | No in the signalling question **(e)** |  |
|  |  | - Unclear - If unclear, please give further details (free text – optional) | If no information is implied | No information in the signalling question **(f)** |  |
|  | 1. If yes or unclear has been answered for question 1, were any participants aware of their assigned intervention during the trial? | - Yes |  | Yes in the signalling question **(g)** |  |
|  |  | - No |  | No in the signalling question **(h)** |  |
|  |  | - Unclear   If unclear, please give further details (free text – optional) | If no information is implied | No information in the signalling question **(i)** |  |
| 2.2. Were carers and trial personnel aware of participants' assigned intervention during the trial? | 1. Were any people who had interactions with the participants (i.e. nurses, doctors, family, trial personnel) aware of participants' assigned intervention during the trial? | - Yes |  | Yes in the signalling question **(j)** | One to one with the signalling question  Yes: **(j)**  No: **(k)**  No information: **(l)** |
|  |  | - No |  | No in the signalling question **(k)** |  |
|  |  | - Unclear   If unclear, please give further details (free text – optional) | If no information is implied | No information in the signalling question **(l)** |  |
| 2.3. If Y/PY/NI to 2.1 or 2.2: Were there deviations from the intended intervention beyond what would be expected in usual practice? | 1. Was there evidence to suggest that there were strong deviations from the intended control condition? | - Yes |  | Yes in the signalling question **(m)** | Just looking at Question 6, one to one with the signalling question  Yes: **(p)**  No: **(q)**  No information: **(r)**  Question 5 is for extra information. |
|  |  | - No |  | No in the signalling question **(n)** |  |
|  |  | - Unclear - If unclear, please give further details (free text – optional) | If no information is implied | No information in the signalling question **(o)** |  |
|  | 1. Was there evidence to suggest that the intervention was not delivered as expected? | - Yes |  | Yes in the signalling question **(p)** |  |
|  |  | - No |  | No in the signalling question **(q)** |  |
|  |  | - Unclear - If unclear, please give further details (free text – optional) | If no information is implied | No information in the signalling question **(r)** |  |
| 2.4. If Y/PY to 2.3: Were these deviations from intended intervention unbalanced between groups *and* likely to have affected the outcome? | 1. If ‘yes’ is answered in question 5 or question 6, Were these deviations from intended intervention unbalanced between groups *and* likely to have affected the outcome? | - Yes |  | Yes in the signalling question **(s)** | One to one with the signalling question  Yes: **(s)**  No: **(S)**  No information: **(t)** |
|  |  | - No |  | No in the signalling question **(S)** |  |
|  |  | - Unclear - If unclear, please give further details (free text – optional) | If no information is implied | No information in the signalling question **(t)** |  |
| 2.5a Were any clusters analysed in a group different from the one to which they were assigned? | 1. For the primary outcome, were any **clusters** analysed in an assigned intervention different from the one to which they were assigned to? | - Yes |  | Yes in the signalling question **(u)** | One to one with the signalling question  Yes: **(u)**  No: **(U)**  No information: **(v)** |
|  |  | - No |  | No in the signalling question **(U)** |  |
|  |  | - Unclear - If unclear, please give further details (free text – optional) | If no information is implied | No information in the signalling question **(v)** |  |
| 2.5b Were any participants analysed in a group different from the one to which their original cluster was randomized? | 1. For the primary outcome, were any **participants** analysed in an assigned intervention different from the one to which they were assigned to? | - Yes |  | Yes in the signalling question **(w)** | One to one with the signalling question  Yes: **(w)**  No: **(W)**  No information: **(x)** |
|  |  | - No |  | No in the signalling question **(W)** |  |
|  |  | - Unclear - If unclear, please give further details (free text – optional) | If no information is implied | No information in the signalling question **(x)** |  |
| 2.6 If Y/PY/NI to 2.5: Was there potential for a substantial impact (on the estimated effect of intervention) of analysing participants in the wrong group? | 1. If yes or unclear answered in question 8, were more than about 5% of **clusters** analysed in an arm different from the one to which they were assigned to? | - Yes |  | Yes in the signalling question **(y)** | Just using Question 11, one to one with the signalling question  Yes: **(z)**  No: **(Z)**  No information: **(zZ)**  Question 9 is for extra detail. |
|  |  | - No |  | No in the signalling question **(Y)** |  |
|  |  | - Unclear - If unclear, please give further details (free text – optional) | If no information is implied | No information in the signalling question **(yY)** |  |
|  | 1. If yes, or unclear answered in question 10, were more than about 5% of **participants** analysed in an assigned intervention different from the one to which they were assigned to? | - Yes |  | Yes in the signalling question **(z)** |  |
|  |  | - No |  | No in the signalling question **(Z)** |  |
|  |  | - Unclear   If unclear, please give further details (free text – optional) | If no information is implied | No information in the signalling question **(zZ)** |  |

|  | | | | | |
| --- | --- | --- | --- | --- | --- |
| **RoB2.0 Signalling questions** | **Data extraction form questions** | **Data extraction answer** | **Comment** | **Standalone what this indicates in signalling question** | **Signalling question answer (given combinations of possible answers)** |
| 3.1a Were outcome data available for  all, or nearly all, clusters randomized? | 1. For the primary outcome, did more than approximately 90% of randomised **clusters** contribute to the **primary analysis**? | - Yes |  | Yes in the signalling question **(a)** | For Question 1: one to one with the signalling question  Yes: **(a)**  No: **(b)**  No information: **(c)**  Question 2 if providing extra detail |
|  |  | - No |  | No in the signalling question **(b)** |  |
|  |  | - Unclear - If unclear, please give further details (free text – optional) | If implied there was no information | No information in the signalling question **(c)** |  |
|  | 1. For the primary outcome, what is the percentage of **clusters** that contributed to the **primary analysis**? (Optional) | - % (range from 0 to 100 inclusive) |  |  |  |
| 3.1b Were outcome data available for all, or nearly all, participants within clusters? | 1. For the **primary outcome**, were outcome data available for more than approximately 90% of **participants**? | - Yes |  | Yes in the signalling question **(d)** | For Question 3 : one to one with the signalling question  Yes: **(d)**  No: **(e)**  No information: **(f)**  Question 4 provides extra detail. |
|  |  | - No |  | No in the signalling question **(e)** |  |
|  |  | - Unclear - If unclear, please give further details (free text – optional) | If implied there was no information | No information in the signalling question **(f)** |  |
|  | 1. For the primary outcome, what is the percentage of **participants** that contributed to the **primary analysis**? (Optional) | - % (range from 0 to 100 inclusive) |  |  |  |
| 3.2 If N/PN/NI to 3.1: Are the proportions of missing outcome data and reasons for missing outcome data similar across intervention groups? | 1. If unclear or no in question 1, are the **proportions** of missing **primary** outcome data similar across control and intervention conditions for **clusters**? | - Yes |  | Yes in the signalling question **(g)** | **Yes:**  If Question 5 and Question 6 both state yes, this indicates **yes** in the signalling question **(g)** and **(j)**  If Question 7 and Question 8 both state yes, this indicates **yes** in the signalling question **(m)** and **(p)**  **No:**  Any combination of Yes/No or No/No, this for either sets of Qs (Question5 & Question 6; Question 7 & Question 8) this indicates No.  **No information:**  If there is anything given as no information in these pairs of Qs then this should be mapped as No information **(i)** or **(l)** or **(o)** or **(r)** |
|  |  | - No |  | No in the signalling question **(h)** |  |
|  |  | - Unclear - If unclear, please give further details (free text – optional) |  | No information in the signalling question **(i)** |  |
|  | 1. If unclear or no in question 1, are the **reasons** for missing **primary** outcome data similar across control and intervention conditions for **clusters**? | - Yes |  | Yes in the signalling question **(j)** |  |
|  |  | - No |  | No in the signalling question **(k)** |  |
|  |  | - Unclear - If unclear, please give further details (free text – optional) |  | No information in the signalling question **(l)** |  |
|  | 1. If unclear or no in question 3, are the **proportions** of missing **primary** outcome data similar across control and intervention conditions for **participants**? | - Yes |  | Yes in the signalling question **(m)** |  |
|  |  | - No |  | No in the signalling question **(n)** |  |
|  |  | - Unclear - If unclear, please give further details (free text – optional) |  | No information in the signalling question **(o)** |  |
|  | 1. If unclear or no in question 3, are the **reasons** for missing **primary** outcome data similar across control and intervention conditions for **participants**? | - Yes |  | Yes in the signalling question **(p)** |  |
|  |  | - No |  | No in the signalling question **(q)** |  |
|  |  | - Unclear   If unclear, please give further details (free text – optional) |  | No information in the signalling question **(r)** |  |
| 3.3 If N/PN/NI to 3.1: Is there evidence that results were robust to the presence of missing outcome data? | 1. If unclear or no in questions 1 or 3, how were missing data handled in the **primary** analysis? | - Multiple imputation |  | **(s)** | **Yes:**  For Question 10, if MI, CC, Sensitivity analysis was answered, i.e. **(w)** and Question 11 says the results are similar then **yes** should be answered  **No:**  For Question 10, if MI, CC, Sensitivity analysis was answered, i.e. **(w)** and Question 11 says the results are NOT similar then **no** should be answered  If Question 10 states no other analysis this should be mapped to **No (v)**  **No information:**  If Question 11 is unclear then this will map to no information in the signalling question. **(z)**  Question 9 provides extra detail. |
|  |  | - Complete case |  | **(S)** |  |
|  |  | - Other - If other, Please give further details (Free text- compulsory to answer) |  | **(t)** |  |
|  |  | - Unclear - If unclear, please give further details (free text – optional) |  | **(u)** |  |
|  | 1. For the primary outcome, were other analysis reported, if so what was reported? | - No other analysis reported |  | **(v)** |  |
|  |  | - Multiple imputation - Complete case - Sensitivity analysis |  | **(w)** |  |
|  |  | - Other - If other, Please give further details (Free text- compulsory to answer) |  | **(W)** |  |
|  |  | - Unclear - If unclear, please give further details (free text – optional) |  | **(x)** |  |
|  | 1. If ‘no other analysis reported’ in question 10 do not answer this question, were the results between the primary analysis and these sensitivity analyses for the primary outcome similar? | - The results were similar |  | **(y)** |  |
|  |  | - The results were not similar |  | **(Y)** |  |
|  |  | - Unclear - If unclear, please give further details (free text – optional) |  | **(z)** |  |

| **RoB2.0 Signalling questions** | **Data extraction form questions** | **Data extraction**  **answers** | **Comment** | **Standalone what this indicates in the signalling question** | **Signalling question answer (given combinations of possible answers)** |
| --- | --- | --- | --- | --- | --- |
| 4.1a Were outcome assessors aware that a trial was taking place? | 1. Were the primary outcome assessors aware that a trial was taking place? | - Yes |  | Yes in the signalling question **(a)** | One to one with the signalling question  Yes: **(a)**  No: **(b)**  No information: **(c)** |
|  |  | - No |  | No in the signalling question **(b)** |  |
|  |  | - Unclear - If unclear, please give further details (free text – optional) | If it is implied there is no information or insufficient information available. | No information in the signalling question **(c)** |  |
| 4.1b If Y/PY/NI to 4.1a: Were outcome assessors aware of the intervention received by study participants? | 1. Were the primary outcome assessors aware of the intervention received by the **cluster**? | - Yes |  | Yes in the signalling question **(d)** | **Yes:**  For Question 4 or Question 6, if yes is answered this indicates yes in the signalling question. **(d)** or **(g)**  **No:**  If Question 4 states Yes and Question 6 states No with a valid quote, then No is answered in the signalling question **(d)** and **(h)**  If Question 4 and Question 6 both state No (or combination of No and No information) then No should be answered in the signalling question **(e)** and **(h)** or **(e)** and **(h)**; or **(f)** and **(h)**.  **No information:**  If both Question 4 and Question 6 state unclear then this maps to no information **(f)** and **(i)**.  Query:  If Q4 states Yes and Q6 states No with no quote then the paper needs to be revisited. |
|  |  | - No |  | No in the signalling question **(e)** |  |
|  |  | - Unclear - If unclear, please give further details (free text – optional) | If it is implied there is no information or insufficient information available. | No information in the signalling question **(f)** |  |
|  | 1. If no or unclear to question 4, how were the **cluster** allocations concealed from the primary outcome assessors? Please provide a quote. | Free text – compulsory to fill in |  |  |  |
|  | 1. Were the primary outcome assessors aware of the intervention received by the **participant**? | - Yes |  | Yes in the signalling question **(g)** |  |
|  |  | - No |  | No in the signalling question **(h)** |  |
|  |  | - Unclear - If unclear, please give further details (free text – optional) | If it is implied there is no information or insufficient information available. | No information in the signalling question **(i)** |  |
|  | 1. If no or unclear to question 6, how were the cluster allocations concealed from the primary outcome assessors? Please provide a quote. | Free text – compulsory to fill in |  |  |  |
| 4.2 If Y/PY/NI to 4.1b: Was the assessment of the outcome likely to be influenced by knowledge of intervention received? | 1. Was the outcome subjective or objective? | - Objective |  | No in the signalling question **(j)** | **Yes:**  If Question 2 states Yes or Question 1 states subjective this would indicate Yes in the signalling question. **(n)** or **(k)**  **No:**  If Question 1 states objective and Question 2 states no this indicates No in the signalling question **(j)** and **(n)**  **No information:**  If question 1 and question 2 state unclear this indicated no information **(l)** and **(o)**  Query:  When Question 1 states objective and Question 2 states yes, this would need to be checked back in the paper if this is the case. |
|  |  | - Subjective |  | Yes in the signalling question **(k)** |  |
|  |  | - Unclear - If unclear, please give further details (free text – not compulsory) |  | No information in the signalling question **(l)** |  |
|  | 1. Was the participant the outcome assessor? | - Yes |  | Yes in the signalling question **(m)** |  |
|  |  | - No |  | No in the signalling question **(n)** |  |
|  |  | - Unclear - If unclear, please give further details (free text – optional) |  | No information in the signalling question **(o)** |  |

| **RoB2.0 Signalling questions** | **Data extraction form questions** | **Data extraction**  **answers** | **Comment** | **Standalone what this indicates in the signalling question** | **Signalling question answer (given combinations of possible answers)** |
| --- | --- | --- | --- | --- | --- |
| 5.1 Reported data selected, on the basis of the results, from multiple outcome measurements? | 1. Has the primary outcome been pre-specified? | - Yes |  | No in the signalling question **(a)** | **No:**  Question 1 and Question 2 stating Yes indicates No in signalling question  **Yes:**  Question 1 or Question 2 stating No or unclear indicates Yes in the signalling question.  **No information:**  If both Question 1 and Question 2 states no information then this indicates no information in the signalling question |
|  |  | - No |  | Yes in the signalling question **(b)** |  |
|  |  | - Unclear - If unclear, please give further details (free-text – optional) |  | No information in the signalling question **(c)** |  |
|  | 1. For the primary outcome only, was an assessment time specified? | - Yes |  | No in the signalling question **(d)** |  |
|  |  | - No |  | Yes in the signalling question **(e)** |  |
|  |  | - Unclear - If unclear, please give further details (free text – optional) |  | No information in the signalling question **(f)** |  |
| 5.2 Reported data selected, on the basis of the results, from multiple analyses of the data? | 1. Was it detailed whether the **primary outcome** results would be reported as adjusted or unadjusted (except for clustering)? | - Yes |  | No in the signalling question **(g)** | **Yes:**  All questions state they had outline pre-specified methods – **(g)** and **(j)** and **(m)** and **(p)** and [**(s)** or **(S)**]  **No:**  Any of these questions state no pre-specified methods – **(h)** or **(k)** or **(n)** or **(q)** or **(t)**  **No information:**  If all questions state no information – **(i)** and **(l)** and **(o)** and **(r)** and **(u)** |
|  |  | - No |  | Yes in the signalling question **(h)** |  |
|  |  | - Unclear - If unclear, please give further details (free text – optional) |  | No information in the signalling question **(i)** |  |
|  | 1. For the primary outcome, was it detailed that the analysis would account for clustering? | - Yes the analysis planned accounted for clustering |  | No in the signalling question **(j)** |  |
|  |  | - No the analysis planned did not account for clustering |  | Yes in the signalling question **(k)** |  |
|  |  | - Unclear - If unclear, please give further details (free text – optional) |  | No information in the signalling question **(l)** |  |
|  | 1. For the primary outcome, were there pre-specified methods given on how to handle missing data for the primary analysis? | - Yes |  | No in the signalling question **(m)** |  |
|  |  | - No |  | Yes in the signalling question **(n)** |  |
|  |  | - Unclear - If unclear, please give further details (free text – optional) |  | No information in the signalling question **(o)** |  |
|  | 1. Were there details on what scale the primary outcome results would be given on?   Notes: For example, HbA1c can be measured and recorded as a percentage or as mmol/mol. | - Yes |  | No in the signalling question **(p)** |  |
|  |  | - No |  | Yes in the signalling question **(q)** |  |
|  |  | - Unclear - If unclear, please give further details (free text – optional) |  | No information in the signalling question **(r)** |  |
|  | 1. If the primary outcome measure was initially a **continuous variable** but for the purpose of this study it was **dichotomised or categorised**, was it clear what cut point was going to be used? | - Yes |  | No in the signalling question **(s)** |  |
|  |  | - The primary outcome measure was not continuous |  | **(S)** |  |
|  |  | - No |  | Yes in the signalling question **(t)** |  |
|  |  | - Unclear - If unclear, please give further details (free text – optional) |  | No information in the signalling question **(u)** |  |
